# Trade-off between processability and device performance in donor–acceptor semiconductors revealed using discrete siloxane side chains†

**DOI:** 10.1039/d4tc00875h

**Published:** 2024-04-24

**Authors:** Bart W. L. van den Bersselaar, Elisabeth H. W. Cattenstart, Kavinraaj Ella Elangovan, Chen Yen-Chi, Bas F. M. de Waal, Joost van der Tol, Ying Diao, E. W. Meijer, Ghislaine Vantomme

**Affiliations:** a Laboratory of Macromolecular and Organic Chemistry and Institute for Complex Molecular Systems, Eindhoven University of Technology P.O. Box 513 5600MB Eindhoven The Netherlands; b Department of Chemical and Biomolecular Engineering, University of Illinois Urbana-Champaign Urbana Illinois 61801 USA

## Abstract

Donor–acceptor polymeric semiconductors are crucial for state-of-the-art applications, such as electronic skin mimics. The processability, and thus solubility, of these polymers in benign solvents is critical and can be improved through side chain engineering. Nevertheless, the impact of novel side chains on backbone orientation and emerging device properties often remains to be elucidated. Here, we investigate the influence of elongated linear and branched discrete oligodimethylsiloxane (*o*DMS) side chains on solubility and device performance. Thereto, diketopyrrolopyrrole–thienothiophene polymers are equipped with various *o*DMS pendants (PDPPTT-Si_*n*_) and subsequently phase separated into lamellar domains. The introduction of a branching point in the siloxane significantly enhanced the solubility of the polymer, as a result of increased backbone distortion. Simultaneously, the charge carrier mobility of the polymers decreased by an order of magnitude upon functionalization with long and/or branched siloxanes. This work unveils the intricate balance between processability and device performance in organic semiconductors, which is key for the development of next-generation electronic devices.

## Abstract 2

The fine balance between enhanced processability and decreased device performance in donor–acceptor polymeric semiconductors is elucidated using discrete siloxane side chains.

## Graphical abstract

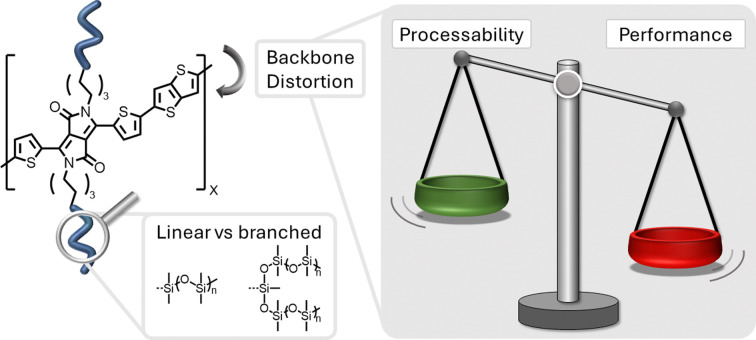

## Introduction

Organic semiconductors are vital for advancing next-generation electronic devices that comprise organic field-effect transistors (OFETs).^1,2^ Hereto, donor–acceptor polymeric semiconductors are particularly suitable due to their ease of processing, mechanical flexibility, lightness and conductivity.^3–6^ Generally, polymeric semiconductors rely on an extended carbon-based π-conjugated system for charge transport.^7^ Commonly, a combination of diketopyrrolopyrrole (DPP) as the acceptor with various thiophene- or selenium-derivatives as the donor moiety is used.^8,9^ However, the crystalline nature of such polymers requires careful molecular design to ensure the material's solubility and, therefore, processability.^10^

Typically, side chains are targeted to tune the solubility of the polymers, ideally without affecting the electronic properties of the main chain.^11–13^ However, recent work showed that the chemical composition of the side chain cannot only benefit the processability, but also the electrochemical features of the material.^14^ Moreover, side chains are an effective tool to improve long-range order, such as lamellar domains, that are often observed in diketopyrrolopyrrole–thienothiophene copolymers.^15,16^ To expand the toolbox of available side chains, the group of Zhenan Bao introduced a short oligodimethylsiloxane (*o*DMS) side chain on a isoindigo-based polymer as a substitute for conventional (branched) alkanes.^17^*o*DMS has a similar cross-sectional area to branched alkanes but induces stronger phase separation between the backbone and the side chains.^18^ Thereby, the π-stacking distance is decreased and subsequently an increase in hole mobility is observed.^19^ However, the use of *o*DMS side chains in literature is limited to the aforementioned trimer or the heptamer.^18,20^ Recently, our group and others have shown that discrete *o*DMS (*Đ* < 1.00001) can greatly enhance the phase separation and nanoscale ordering of materials.^21–24^ Yet, to the best of our knowledge, D–A polymers with longer and/or branched *o*DMS side chains have not been reported. In previous years, we have reported on the synthesis of elongated and branched *o*DMS as side chains for supramolecular polymers.^25,26^ We envision that the use of these side chains in semicrystalline D–A polymers will increase their processability. Furthermore, we aim to elucidate the effect of such pendants on the electronic properties of the semiconductor in an OFET device.

**Fig. 1:**
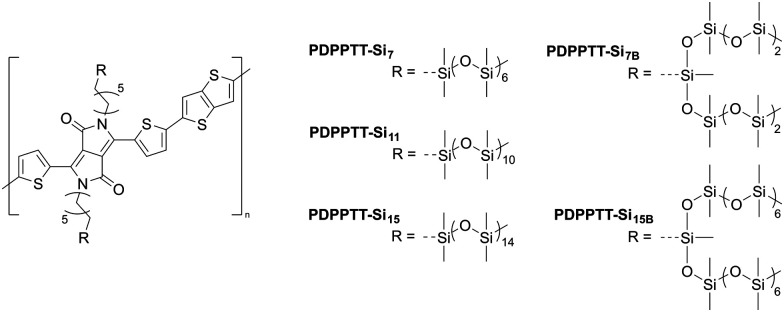
Chemical structures of the polydiketopyrrolopyrrole–thienothiophene derivatives with various *o*DMS side chains (PDPPTT-Si_*n*_).

Herein, we report on the design, synthesis, and characterization of five donor–acceptor copolymers comprising diketopyrrolopyrrole (DPP) and thienothiophene (TT) with various discrete oligodimethylsiloxane (*o*DMS) side chains (Fig. 1). We investigated the effect of linear (Si_7_, Si_11_ and Si_15_) and branched (Si_7B_ and Si_15B_) discrete *o*DMS side chains on the device properties of D–A polymers (PDPPTT-Si_*n*_). Solubility and aggregation of PDPPTT-Si_*n*_ were probed using ultraviolet-visible light spectroscopy (UV-Vis) in various solvents. Thereafter, PDPPTT-Si_*n*_ were investigated with polarized optical microscopy (POM), atomic force microscopy (AFM), medium/wide angle X-ray scattering (MAXS/WAXS), grazing incidence X-ray diffraction (GIXD), differential scanning calorimetry (DSC), thermogravimetric analysis (TGA) and dynamic mechanical (thermal) analysis (DMA/DMTA) to obtain detailed information about their morphological, thermal, and mechanical properties. Finally, the device performance of PDPPTT-Si_*n*_ was probed by fabricating bottom-gate-top-contact (BGTC) OFETs and recording their transfer- and output-curves. Subsequently, their charge carrier mobilities, on/off ratios and threshold voltages were analyzed.

## Results and discussion

### Synthesis of PDPPTT-Si_*n*_

A library of discrete *o*DMS chains was prepared to study the effect of adaptation in the side chain on the processability and device performance of D–A semiconductors. Linear and branched derivatives Si_7_H, Si_11_H, Si_15_H and Si_7B_H and Si_15B_H were synthesized following literature protocol.^25,26^ From either commercially available Si_3_H or previously synthesized Si_7_H, the respective hydroxysiloxanes were formed using Pd/C in a dioxane/water system. These were subsequently mixed with dichloromethylsilane in pyridine/toluene to yield the desired branched side chains on a multigram scale in moderate to good yield (65–82%).

The polymers were synthesized using a protocol inspired by literature (Supporting Information 2, ESI†).^20^ First, the DPP precursor was reacted with 6-bromo-1-hexene for 72 h, *versus* the reported protocol of 24 h, resulting in a significant increase in yield (49% *versus* 27%). Hereafter, the acceptor was brominated using NBS and equipped with the desired *o*DMS in a platinum-catalyzed hydrosilylation reaction. Finally, the decorated DPP moiety was reacted with thienothiophene (TT) *via* a Stille-coupling in toluene, yielding all PDPPTT-Si_*n*_ as green solids. The polymers were consecutively extracted with ethanol, acetone, cyclohexane, and chloroform using a Soxhlet set-up to remove low *M*_w_ impurities. During these purification steps, it was already observed that PDPPTT-Si_7B_ and PDPPTT-Si_15B_ showed enhanced solubility, in line with previous research on linear and branched alkane side chains.^11^ The resulting molecular weight and dispersity were estimated using gel permeation chromatography in ortho-dichlorobenzene (*o*-DCB) at 140 °C and are displayed in Table 1 and Fig. S1 (ESI†). PDPPTT-Si_7_ and PDPPTT-Si_7B_ showed the largest *M*_w_ of all PDPPTT-Si_*n*_ which has been reported to have an influence on the mechanical and device properties of the semiconductor.^27^

**Table 1:** Bulk properties of PDPPTT-Si_*n*_

Polymer	*M*_n_ (kDa)	*M*_w_ (kDa)	*Đ* a (—)	*d*_lam_ b (nm)	*d*_π–π_ b (nm)	*T*_d_ c (°C)	Young's modulus^d^ (MPa)	Fracture strain^e^ (%)
PDPPTT-Si_7_	111	347	3.1	3.4	0.35	408	1.3	6.9
PDPPTT-Si_11_	42	141	3.4	4.1	0.35	389	0.26	8.1
PDPPTT-Si_15_	33	119	3.6	4.5	0.35	394	0.17	4.4
PDPPTT-Si_7B_	69	235	3.4	2.8	n.o.	408	0.66	14.5
PDPPTT-Si_15B_	55	116	2.3	3.2	n.o.	397	n.a.^f^	n.a.^f^

a Determined from GPC in *o*-DCB at 140 °C using polystyrene standards. The tails in the chromatograms were excluded from the integration (Fig. S1, ESI).

b Spacing determined from the MAXS spectra using *d* = 2π/*q* (Fig. 3D).

c Temperature at which 5% weight loss was observed in TGA (Fig. S13, ESI).

d Young's modulus was calculated from the slope of the stress–strain curve at strain <0.5% (Fig. 4B).

e Fracture strain was determined from the onset of failure in the material.

f Due to the waxy material, no free-standing films were obtained.

### UV-Vis studies of PDPPTT-Si_*n*_

**Fig. 2:**
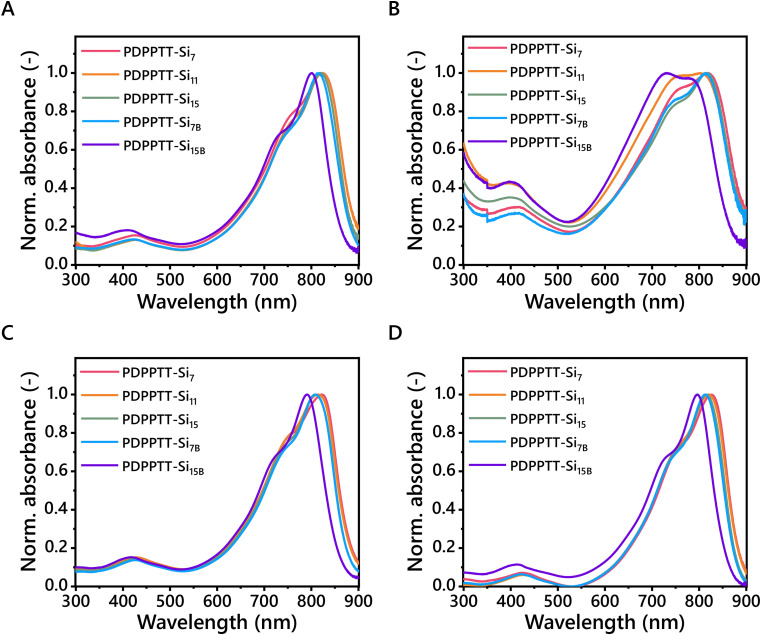
UV-Vis spectra of PDPPTT-Si_*n*_ in (A) CHCl_3_, (B) *o*-DCB, (C) methylcyclohexane and (D) toluene (*l* = 1 mm, *c* = 0.08 mg mL^−1^).

The solubility, and therefore processability, of PDPPTT-Si_*n*_ was subsequently probed by recording their UV-Vis absorption spectra (Fig. 2). All PDPPTT-Si_*n*_ showed the presence of three characteristic absorption bands. Firstly, a local maximum around 400 nm was observed, which was previously correlated to delocalized excitonic π–π* transitions in the conjugated backbone.^28,29^ Besides, the presence of intramolecular charge transfer (ICT) between donor- and acceptor moieties was evident from the peak above 700 nm (*λ*_ICT_). The observed splitting of this high-wavelength band in all solvents is indicative of a high degree of ordering in the backbone as well as the formation of pre-aggregates.^30,31^ The shoulder at 730 nm is attributed to the 0-1 transition. Similarly, the lowest energy band was identified as the 0-0 transition. The presence of such vibronically structured bands is indicative of planarization of the backbone to a certain degree, where a decrease of the ratio *A*_0-0_/*A*_0-1_ points towards enhanced distortion from the planar ordered state.^32^ Such a relative increase of the intensity of the 0-1 peak with respect to the 0-0 peak was clearly observed for PDPPTT-Si_7B_ in CHCl_3_ and for PDPPTT-Si_11_ and PDPPTT-Si_15B_ in *o*-DCB. This observation, combined with the blue shift of the low-energy bands in general, infers that the displayed trend of solubility increase in PDPPTT-Si_*n*_ in CHCl_3_ stems from increased backbone distortion (Table 2).

**Table 2:** Spectroscopic and device properties of PDPPTT-Si_*n*_

Polymer	*λ*_max_ a (nm)	*E*_g,SWV_ (eV)	HOMO (eV)	LUMO (eV)	Mobility (cm^2^ V^−1^ s^−1^)	On/off ratio (—)	Threshold voltage (V)
PDPPTT-Si_7_	823	1.53	−5.28	−3.75	0.18	1.29 × 10^4^	0.75
PDPPTT-Si_11_	822	1.56	−5.28	−3.72	0.09	2.00 × 10^4^	1.11
PDPPTT-Si_15_	820	1.59	−5.3	−3.71	0.01	0.25 × 10^4^	4.45
PDPPTT-Si_7B_	815	1.60	−5.34	−3.74	0.04	0.39 × 10^4^	5.52
PDPPTT-Si_15B_	800	1.55	−5.31	−3.76	n.a.^b^	n.a.^b^	n.a.^b^

a Values determined from samples in CHCl_3_.

b No successful OFET device was fabricated of this polymer.

To investigate whether this effect was also apparent in other solvents, the solubility of PDPPTT-Si_*n*_ was subsequently screened in *o*-DCB, methylcyclohexane and toluene (Fig. 2B–D). There, similar hypsochromic shifts were observed at room temperature for PDPPTT-Si_*n*_ in all solvents. *o*-DCB is a solvent known to dissolve such polymers well, which we recognized by the different shape of the low-energy band for PDPPTT-Si_11_ and PDPPTT-Si_15B_ (Fig. 2B). Remarkably, PDPPTT-Si_15B_ showed a higher relative intensity for the 0-1 peak compared to the 0-0 peak in *o*-DCB at room temperature, which was only observed at elevated for other PDPPTT-Si_*n*_ (Fig. S2–S6, ESI†). Thus, the decreased planarity of the polymeric chain enables the formation of predominantly disordered structures already at room temperature for PDPPTT-Si_15B_ in *o*-DCB. Strikingly, complete merger of the 0-0 and 0-1 peaks was observed at elevated temperature in *o*-DCB, indicating a molecularly dissolved state (Fig. S6D, ESI†). Hence, these results demonstrate that the improved solubility of all PDPPTT-Si_*n*_ in various solvents is dominated by the increased backbone distortion.

### Bulk studies of PDPPTT-Si_*n*_

**Fig. 3:**
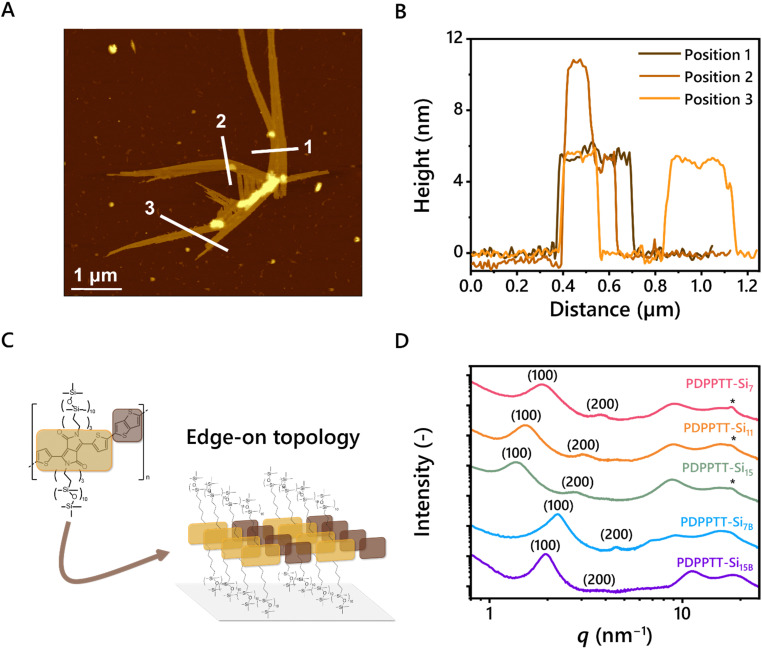
Investigation of PDPPTT-Si_n_ in bulk. (A) Tapping mode AFM height image (5 × 5 μm) of spin coated solution of PDPPTT-Si_11_ (8 × 10^−2^ mg mL^−1^ in CHCl_3_, deposited on freshly cleaved mica). (B) Extracted height profiles of the PDPPTT-Si_11_ fibers along the white lines. (C) Proposed molecular packing of the edge-on topology of the fibers when deposited on mica. (D) MAXS/WAXS scattering profiles of PDPPTT-Si_*n*_ where *q* is the principal scattering peak and * indicates the [010]-reflection originating from π–π interactions.

The processability of the aggregated species in dilute solutions was explored by spin coating solutions of PDPPTT-Si_11_ in CHCl_3_ (8 × 10^−2^, 8 × 10^−3^ and 8 × 10^−4^ mg mL^−1^) on freshly cleaved mica and subsequent analysis with AFM (Fig. S7A–D, ESI†). Intriguingly, elongated structures were observed that reached up to 10 μm for the most concentrated solutions (Fig. 3A). The width of the structure revealed that the fibers consist of several aligned polymer chains. Interestingly, the measured height was consistent throughout the sample, which corresponds to the calculated side chain end-to-end distance of one DPPTT-Si_11_ moiety (5.4 nm, Fig. 3A and B). A few exceptions of bright spots were observed and attributed to overlapping bundles (for example in line 2, Fig. 3B). These results show that the polymers are oriented in the desired edge-on orientation (Fig. 3C), which is beneficial for charge carrier mobility in OFET devices.^33^ Besides proper orientation, the presence of long-range order is an important requirement for efficient charge carrier transport. Using POM, clear birefringence was demonstrated with all PDPPTT-Si_*n*_ under polarized light at room temperature, hinting at the presence of nanoscale order in the material (Fig. S8–S12, ESI†). The nanostructure of PDPPTT-Si_*n*_ was studied in more depth using medium- and wide-angle X-ray scattering (MAXS and WAXS, Fig. 3D). Lamellar morphologies with different domain spacings were observed for all PDPPTT-Si_*n*_ (*d*_lam_, Table 1), indicated by reflections at integer numbers from the principal scattering peak (100, 200, 300, …). In these 2D nanostructures, the *o*DMS side chains are partially intercalated as determined from the non-linear increase in domain spacing from PDPPTT-Si_7_ to PDPPTT-Si_15_. Nevertheless, increasing the length of the *o*DMS side chains only slightly affects the order in the material, exemplified by the diminished intensity of the secondary and tertiary reflection peaks from PDPPTT-Si_7_ to PDPPTT-Si_15_. Moreover, the polymers with linear *o*DMS side chains showed a clear reflection at *q* = 18.2 nm^−1^, corresponding to the intramolecular π-stacking distance typically observed for organic semiconductors of 0.35 nm.^34^ However, the intensity of the π-stacking peak decreased with increasing length of the *o*DMS side chain and disappeared completely in PDPPTT-Si_7B_ and PDPPTT-Si_15B_. These results corroborate the findings from the UV-Vis spectroscopy studies shown in Fig. 2, as the longer and branched *o*DMS side chains impose more backbone distortion on the polymer which consequently results in less efficient packing, which is most pronounced in polymers with branched side chains.

### Thermal and mechanical stability of PDPPTT-Si_*n*_

**Fig. 4:**
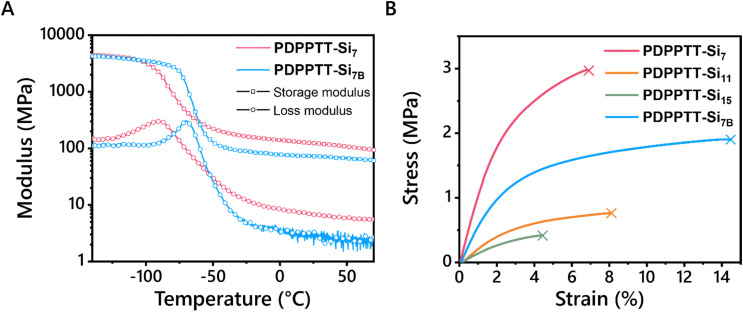
Mechanical properties of PDPPT-Si_*n*_. (A) DMA traces of PDPPTT-Si_7_ and PDPPTT-Si_7B_ showing storage and loss moduli as a function of temperature. A heating rate of 3 °C min^−1^ at a frequency of 1 Hz and a strain of 0.1% were applied. (B) Stress–strain curves for PDPPTT-Si_7_, PDPPTT-Si_11_, PDPPTT-Si_15_, and PDPPTT-Si_7B_ obtained from tensile tests on free-standing films.

Application of such polymers as stretchable semiconductors require the presence of their glass transition temperature (*T*_g_) far below room temperature. Hence, thermal transitions of PDPPTT-Si_*n*_ were explored using differential scanning calorimetry (DSC, Fig. S13A, ESI†). No melting or crystallization transitions were seen below 250 °C, which is common for rigid conjugated polymers.^4^ Moreover, no *T*_g_'s were observed within the limits of the DSC machine (−70 °C to 250 °C). Therefore, PDPPTT-Si_7_ and PDPPTT-Si_7B_ were characterized using dynamic mechanical thermal analysis (DMTA) to probe the presence of a *T*_g_ at temperatures below −70 °C (Fig. 4). Here, PDPPTT-Si_7_ and PDPPTT-Si_7B_ were selected since increasing side chain length is known to decrease the *T*_g_ of organic semiconductors and thus these two polymers were expected to show the lowest *T*_g_.^35^ As observed from the tan(*δ*) curve (Fig. S13B, ESI†), peaks were observed at −74 °C and −62 °C for PDPPTT-Si_7_ and PDPPTT-Si_7B_, respectively. Based on previous literature, this transition is assigned to the relaxation of the *o*DMS side chains (*T*_*γ*_).^36^ We rationalize that the difference of 12 °C stems from the greater flexibility in PDPPTT-Si_7_ compared to PDPPTT-Si_7B_ due to the presence of the branching point in the latter system.^37^

Next, the thermal stability of the polymers at elevated temperature was studied by investigating their decomposition. Hereto, TGA measurements were executed, and the 5% thermal weight loss temperatures (*T*_d_) were determined (Fig. S13C, ESI† and Table 1). For all polymers, these values ranged from 389 to 408 °C, similar to previously reported polymers.^20^ In addition, no significant differences were observed with the introduction of longer and/or branched side chains. Moreover, tensile tests were performed to check the influence of the side chains on the mechanical properties of PDPPTT-Si_*n*_ materials (Fig. 4B). Free-standing films were successfully prepared by solvent-casting PDPPTT-Si_*n*_ from CHCl_3_ in a Teflon mold, except for PDPPTT-Si_15B_ due to its waxy nature. The Young's modulus was determined from the slope in the linear regime (strain < 0.5%). The highest Young's modulus was observed for PDPPTT-Si_7_ (1.3 MPa), after which it decreased with increasing siloxane length. The eightfold decrease in modulus between PDPPTT-Si_7_ and PDPPTT-Si_15B_ is significantly larger than previously reported changes in modulus based on molecular weight.^27^ Moreover, the change observed by doubling the *o*DMS length is similar to the reported change upon doubling the length of an alkane sidechain in organic semiconductors.^38^ Hence, we conclude that the decreasing stiffness is a result of increasing side chain length. The Young's modulus of PDPPTT-Si_7B_ (0.66 MPa) was observed to be lower than PDPPTT-Si_7_ (1.3 MPa), yet higher than PDPPTT-Si_11_ (0.26 MPa). This difference shows that the length of the side chain is of greater relative importance for the mechanical properties of the polymer than the branching of the side chain. Overall, the Young's moduli of PDPPTT-Si_*n*_ are approximately two orders of magnitude lower than comparable polymers equipped with alkane side chains, which can be attributed to the plasticizing effect of *o*DMS. Besides, polymeric semiconductors often have a fracture strain below 10%, due to their crystalline nature.^39^ Linear PDPPTT-Si_*n*_ displayed fracture strains between 4.4 and 8.1% (Fig. 4B), while PDPPTT-Si_7B_ showed a significant increase in fracture strain (14.5%). The loss of crystallinity in PDPPTT-Si_*n*_ due to the aforementioned backbone distortion thus decreases the Young's modulus of the material, whilst simultaneously increasing its fracture strain. These mechanical properties will result in reduced interfacial stress in devices and hence are desirable for applications in flexible materials.

### Device performance of PDPPTT-Si_*n*_

Finally, to test the device performance of PDPPTT-Si_*n*_, their HOMO/LUMO levels must correspond with the work function of the electrode. Thereto, HOMO/LUMO levels were probed using square wave voltammetry (SWV) on thin films (Table 2, Fig. S14 and S15, ESI†). Previous research reported a slight increase of the LUMO-level with increasing side chain length.^20^ Contrarily, PDPPTT-Si_*n*_ exhibited similar electrochemical energy levels, unaffected by the length or branching of the side chains. The HOMO-level allows for injection of electrons from a silver electrode, necessary for fabrication of the OFET.

**Fig. 5:**
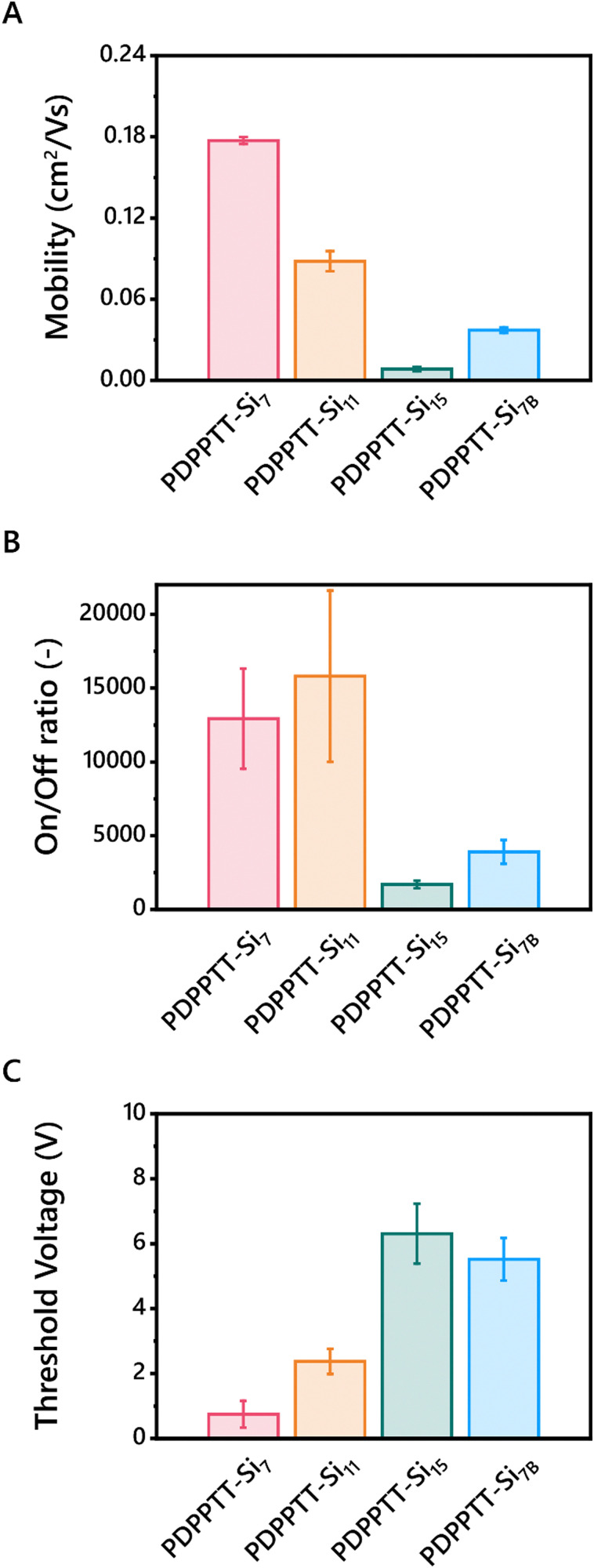
Charge carrier mobilities (A), on/off ratios (B) and threshold voltages (C) of BGTC OFETs fabricated from PDPPTT-Si_*n*_.

Subsequently, bottom-gate-top-contact OFETs were fabricated from blade coated PDPPTT-Si_7_, PDPPTT-Si_11_, PDPPTT-Si_15_ and PDPPTT-Si_7B_ thin films. Here, PDPPTT-Si_15B_ is excluded as we were unable to fabricate working OFET devices from this polymer due to the limited amount of material available. The device preparation and experimental details are listed in the ESI.† By varying the printing speed, the thickness of the semiconducting layer was controlled (Fig. S16, ESI†) and devices with a thickness of 140 ± 20 nm were analyzed. All polymers were processed into working devices and their transfer and output curves were recorded (Fig. S17, ESI†). Based on the reported literature, the highest *M*_w_PDPPTT-Si_*n*_ were expected to give lower mobilities due to the presence of entanglements.^27^ Contrarily, maximum charge carrier mobilities of 0.18 cm^2^ V s^−1^, 0.09 cm^2^ V s^−1^, 0.01 cm^2^ V s^−1^ and 0.04 cm^2^ V s^−1^ for PDPPTT-Si_7_, PDPPTT-Si_11_, PDPPTT-Si_15_ and PDPPTT-Si_7B_ respectively, were extracted (Table 2 and Fig. 5A). We hypothesize that careful optimization of the sample preparation and annealing conditions can significantly increase these mobility values further though, as PDPPTT polymers with alkyl side chains have been reported to have mobilities up to 10 cm^2^ V s^−1^.^40^ Nevertheless, the observed mobility for PDPPTT-Si_7_ was similar to siloxane decorated polymers reported previously.^20^ Contrary to these reported PDPPTT-Si_*n*_ that showed no change in mobility upon functionalization with longer siloxanes, we observed a large decrease in device performance. The decreased device performance was also shown in the on/off ratio, where a significant decline was observed upon elongation or introduction of branching of the *o*DMS side chains (Fig. 5B). Finally, PDPPTT-Si_7_ was shown to exhibit a lower threshold voltage compared to other PDPPTT-Si_*n*_ (Fig. 5C). We hypothesize that the decrease in *V*_TH_ originates from a combination of a lower lying HOMO, as well as the diminished π-stacking with increasing side chain length and the introduction of a branching point, leading to the formation of more charge carrier traps as the electron hopping process is less efficient. Notably, the loss in performance between PDPPTT-Si_7_ and PDPPTT-Si_11_ was lower than the observed decrease between PDPPTT-Si_11_ and PDPPTT-Si_15_. We hypothesize that the aforementioned distortion of the backbone decreases the overlap of the π-system, which diminishes efficient electron hopping.

To test this hypothesis, grazing incidence X-ray diffraction (GIXRD) spectra were recorded on printed films of PDPPTT-Si_*n*_ to investigate the presence of π-stacking in the devices (Fig. S18, ESI†). As apparent from the 1D spectra, all PDPPTT-Si_*n*_ display the presence of a peak at 17.7 nm^−1^, indicative of a π-stacking distance of 3.55 Å. However, from the 2D images it was apparent that PDPPTT-Si_7_ and PDPPTT-Si_11_ showed the most intense [010]-reflection, which diminished in PDPPTT-Si_15_ and PDPPTT-Si_7B_ (Fig. S19, ESI†). This decreasing peak intensity follows the measured trend in mobility as shown in Fig. 5A. Additionally, it was observed that all printed PDPPTT-Si_*n*_ adopted a favorable edge-on orientation to the substrate.

We rationalize that these phenomena are the origin of the significant decrease in charge carrier mobility of PDPPTT-Si_7B_ compared to PDPPTT-Si_7_ (Fig. 5). Besides, the decrease in performance was larger (*Δ*_mobility_ = 0.14 *vs.* 0.09 cm^2^ V s^−1^) than observed between PDPPTT-Si_7_ and PDPPTT-Si_11_. Thus, the disruption of the backbone planarity and consequent loss of π-stacking between adjacent polymer chains that were displayed through UV-Vis and X-ray scattering techniques are detrimental for the emerging electronic properties.

## Conclusion

We have demonstrated the design, synthesis, and characterization of PDPPTT semiconductors with long and/or branched discrete *o*DMS. We displayed that these pendants can be applied to increase the solubility in both halogenated as well as more benign solvents, which is beneficial for the processability of these materials. Additionally, 2D lamellar structures of PDPPTT-Si_*n*_ adopt favorable edge-on topologies both in spin-coated and in printed samples. However, both the long-range order and π-stacking were shown to diminish with increasing length and especially branching of the side chain, which is associated with decreased planarity in the polymers. Furthermore, OFET fabrication from PDPPTT-Si_7_, PDPPTT-Si_11_, PDPPTT-Si_15_ and PDPPTT-Si_7B_ revealed the negative influence of backbone distortion on the device performance. We displayed an order of magnitude decrease in charge carrier mobility when longer and/or branched *o*DMS side chains were utilized. Thus, combining UV-Vis absorption, X-ray scattering and charge carrier mobility studies we revealed the trade-off between increased processability yet decreasing OFET performance through the use of long and/or branched siloxane side chains in organic polymeric semiconductors. Therefore, future research towards polymeric semiconductors should carefully tune the side chains of the polymers during design, to control the inherent trade-off between solubility and device performance.

## Conflicts of interest

There are no conflicts to declare.

## Supplementary Material

TC-012-D4TC00875H-s001
