# Supplementary material for: Trade-off between processability and device performance in donor–acceptor semiconductors revealed using discrete siloxane side chains
Source: J Mater Chem C Mater. 2024 Apr 24;12(18):6637–44. doi: 10.1039/d4tc00875h (PMC11079859; doi:10.1039/d4tc00875h)
Supplement: TC-012-D4TC00875H-s001 [file TC-012-D4TC00875H-s001.pdf]

Electronic Supplementary Material (ESI) for Journal of Materials Chemistry C.
This journal is © The Royal Society of Chemistry 2024

Supporting information

Trade-off Between Processability and Device Performance in Donor-Acceptor Semiconductors revealed using Discrete Siloxane Side Chains

Bart W.L. van den Bersselaar,[a] Elisabeth H.W. Cattenstart,[a] Kavinraaj Ella Elangovan,[b] Yen-Chi Chen,[b] Bas F.M. de Waal,[a] Joost van der Tol,[a] Ying Diao,[b] E.W. Meijer[a] and Ghislaine Vantomme,[a]*

[a]Laboratory of Macromolecular and Organic Chemistry and Institute for Complex Molecular Systems, Eindhoven University of Technology P.O. Box 513, 5600MB Eindhoven, The Netherlands

[b]Department of Chemical and Biomolecular Engineering, University of Illinois Urbana-Champaign, Urbana, Illinois 61801, United States of America

*E-mail: g.vantomme@tue.nl

Table of contents

1. **Materials and methods** ...2
2. **Synthesis of PDPPTT-Si$_n$** ...6
3. **Recycle GPC traces of PDPPTT-Si$_n$** ...14
4. **UV-Vis spectra of PDPPTT-Si$_n$ in various solvents** ...15
5. **AFM imaging of PDPPTT-Si$_{11}$** ...18
6. **POM images of PDPPTT-Si$_n$** ...19
7. **Thermal and Mechanical properties of PDPPTT-Si$_n$** ...22
8. **Square wave voltammograms of PDPPTT-Si$_n$** ...23
9. **BGTC OFETs characteristics of PDPPTT-Si$_n$** ...24
10. **NMR spectra** ...27
11. **References** ...38

1. Materials and methods

Linear and branched *o*DMS were synthesized according to literature procedure.[1,2] All other reagents and chemicals were purchased from commercial suppliers at the highest available purity and used without further purification. All solvents were of analytical grade and purchased from Biosolve. Water was demineralized using an EMD Millipore Milli-Q integral Water Purification System. Dry solvents were acquired using MBraun solvent purification system (MB SPS-800). Deuterated chloroform was purchased from Cambridge Isotopes Laboratories.

Thin-layer chromatography (TLC) was performed on precoated 0.25 mm, 60-F_{254} silica gel plates from Merck to follow the conversion of the reactions. A 366 nm ultraviolet lamp was used for visualization. Automatic column chromatography was performed using a Grace Reveleris X2 using Reveleris Silica Flash Cartridges.

Nuclear magnetic resonance (NMR) spectra were acquired using a Bruker 400 MHz Ultrashield spectrometers (400 MHz for ^{1}H NMR, 100 MHz for ^{13}C NMR). Deuterated chloroform was used, and the shift of the trace chloroform (7.26 ppm in ^{1}H NMR and 77.36 in ^{13}C NMR) was used as an internal standard. Chemical shifts (δ) are expressed in ppm values relative to tetramethylsilane (TMS). Peak multiplicity is abbreviated as s: singlet; d: doublet; t: triplet, q: quartet; dd: double doublet; m: multiplet.

Matrix-assisted laser desorption/ionization-time of flight mass spectroscopy (MALDI-TOF-MS) spectra were recorded on a Bruker Autoflex Speed MALDI-TOF. The used matrices were using α-cyano-4-hydroxycinnamic acid (CHCA) and trans-2-[3-(4-tert-butylphenyl)-2-methyl-2-propenylidene]malononitrile (DCTB). All samples were dissolved in chloroform. All samples were prepared in 1 mg mL^{-1} solutions.

Fourier-Transform Infrared (FT-IR) spectra were obtained using a Perkin Elmer Spectrum Two FT-IR spectrometer at room temperature. The spectra were measured from 400 cm^{-1} to 4000 cm^{-1} and averaged over 16 scans.

Molecular weight distributions of the polymers were estimated using GPC in *o*-DCB at 140 °C using polystyrene internal standards on a PL-GPC 120 system using a PL-GEL 10 mm MIXED-C column. The samples were dissolved in *o*-DCB by sonication and then heated before measurements at a concentration of 0.1 mg mL^{-1} for **PDPPTT-Si_7** and **PDPPTT-Si_{7B}** and 1 mg mL^{-1} for **PDPPTT-Si_{11}**, **PDPPTT-Si_{15}**, and **PDPPTT-Si_{15B}**.

Polarized optical microscopy (POM) images were made using a Jenaval polarization microscope with crossed polarizers. The polymers were smeared out as a thin layer on a glass substrate and covered with a second glass substrate.

Bulk small-angle X-ray scattering (SAXS) was performed on an instrument from Ganesha Lab. The flight tube and sample holder are all under vacuum in a single housing, with a GeniX-Cu ultra-low divergence X-ray generator. The source produces X-rays with a wavelength (λ) of 0.154 nm and flux of 1 × 108 ph s^{-1}. Scattered X-rays were captured on a 2-dimensional Pilatus 300K detector with 487 × 619 pixel resolution. The sample-to-detector distance was 0.084 m (WAXS mode) or 0.48 m (MAXS mode). The instrument was calibrated with diffraction patterns from silver behenate.

Grazing-incidence X-ray diffraction (GIXRD) was performed at the complex materials scattering beamline 11-BM at the National Synchrotron Light Source II (NSLS II), Brookhaven National Laboratory with an X-ray wavelength of 0.9184 Å (E_{beam} = 13.5 keV). A Pilatus 800k detector was used for data collection at a 260 mm sample-to-beam detector distance. Samples were measured at four different incidence angles (0.08°, 0.10°, 0.12° and 0.14°) using an automated robotic sample exchanger. Samples were prepared by printing the polymers on OTS treated silicon substrates with a speed of 0.7 mm s^{-1} from a solution of 10 mg mL^{-1} in $CHCl_3$ at 30 °C. The data recorded at 0.10° was used to compare the various samples and the exposure time was 10 s. Each sample was scanned at two in-plane rotation angles (φ) by rotating the substrate with respect to the incidence beam by 0° and 90°. Herein, φ is defined as 0° when the film coating direction is oriented parallel to the incident beam. Data analysis was performed with the software SciAnalysis and GIXSGUI, which included a correction for the polarization of the synchrotron X-ray beam.

Differential Scanning Calorimetry (DSC) was recorded on a Q2000 from TA Instruments. A constant heating and cooling rate of 10 °C min^{-1} were used from −70 °C to 250 °C. To standardize the thermal history such a cycle was executed before recording two heating and cooling cycles.

Thermogravimetric analysis (TGA) was executed on a TGA550 from TA Instruments. The sample was first heated to 100 °C and kept at an isothermal for 30 minutes. Subsequently, the sample was heated from 100 °C to 800 °C at 10 °C min^{-1}, while the weight of the sample was recorded.

Solvent-casted films were measured on a DMA850 (TA Instruments) with a film tension setup. The films were cast from chloroform at a concentration of 60 mg mL^{-1} into a Teflon mold. Before casting the solution, it was sonicated for several hours to ensure the polymers were dissolved. The films were left to dry to air and subsequently dried under vacuum for one night. A temperature ramp from −140 to 80 °C was performed with a heating rate of 3 °C min^{-1} at a frequency of 1 Hz, and a strain of 0.1 % were applied. The storage and loss moduli were recorded as a function of temperature. The glass transition temperature was determined from

the peak maximum in the loss modulus. The stress-strain curves were measured at a displacement of 0 to 25 mm with a ramp rate of 0.5 mm min^{-1}, and a preload force of 0.005 N was applied.

Atomic Force Microscopy (AFM) images were recorded in repulsive tapping mode (phase < 90°) using a Cypher Environmental Scanner (ES) equipped with a closed-cell, a heater-cool stage, and a normal laser diode. Silicon probes (NCSTR-50) with a tip height of 10–15 µm and a radius of < 10 nm were used for all measurements and calibrated using the 'Get Real' function in the Igor Pro software. 20 × 20 µm height images were acquired using a scan rate of 2.0 Hz and 1024 x 1024 pixels. The contrast of the images was further enhanced using first-order plane fit and flattening using Gwyddion v2.60. Samples were prepared by spin coating (800 rpm) 80 µL of solution ($8\cdot10^{-2}$, $8\cdot10^{-3}$, $8\cdot10^{-4}$ mg mL^{-1}) in $CHCl_3$ onto freshly cleaved 1.5 × 1.5 cm^2 sized Mica followed by overnight drying in air.

Variable temperature ultraviolet-visible (VT UV-vis) absorbance spectra were recorded on a Jasco V-750 UV-Vis spectrometer equipped with a PAC-743 multi-cuvette holder and Jasco ETCT-762 temperature controller. Measurements were performed using Quartz cuvettes (Hellma) with a path length of 1 mm. First, a baseline of the corresponding solvent was measured. Samples for VT UV-vis were prepared in a concentration of 0.08 mg mL^{-1} in spectral grade solvents. All samples were sonicated for one hour or until the polymer was completely dissolved to the eye. The samples were cooled from 90 °C to 20 °C with 1 °C min^{-1}. All measurements were performed with a bandwidth of 1.0 nm, a scan speed of 100 nm min^{-1}, and a data interval of 0.1 nm, spanning the UV-Vis range from 300 nm to 900 nm.

All the square wave voltammetry (SWV) measurements were performed in a nitrogen-filled glovebox with a Biologic VSP potentiostat using a three-electrode electrochemical cell. The polymers were deposited on a platinum electrode through dip coating. Another platinum electrode was used as the counter electrode and a silver chloride coated silver rod (Ag/AgCl) was used as a quasi-reference electrode. 0.1 M tetrabutylammonium hexafluorophosphate in acetonitrile was used as the electrolyte solution. Ferrocene/ferrocenium (Fc/Fc^+) was used as an internal reference.

All OFET devices were fabricated in a bottom gate top contact configuration using a meniscus guided printing method followed by deposition of electrode materials by thermal evaporation. The polymers were dissolved in chloroform (99.8% ACS grade, Sigma Aldrich) at a concentration of 10 mg mL^{-1} to make the ink that was used to print the films. The substrate used was an octadecyltrichlorosilane (OTS, Sigma Aldrich) monolayer treated heavily doped Silicon wafer (n-doped) with a thermally grown 300 nm SiO_2 layer which functioned as the bottom gate and dielectric layer respectively. A smaller OTS treated Si wafer piece with 300

nm of SiO_2 was used as the printing blade when depositing the polymer film. The blade and the substrates were rinsed with toluene, acetone and isopropyl alcohol and dried with nitrogen before deposition of the organic materials. The blade height was set at 100 µm and the blade angle was set at 7 degrees. The printing speed of the blade was varied between 0.1 and 0.7 mm s^{-1} to obtain films of varying thicknesses, the substrate temperature was maintained at 30 °C and 10 µL of solution was used to deposit each film. For OTS functionalization of the substrate and blade, precleaned SiO_2 wafer with 300 nm oxide layer was first plasma treated and immersed in trichloroethylene (anhydrous > 99%, Sigma Aldrich) solution of OTS (0.2 vol%) at room temperature for 20 min. The wafer was then rinsed with toluene and isopropanol followed by baking at 120 °C for 20 min and finally performing ultrasonication in toluene for 3 min. Electrodes were fabricated by thermally evaporating 8 nm of Molybdenum trioxide (MoO_3) at a rate of 0.05 Å s^{-1} followed by 45 nm of silver at a rate of 0.5 Å s^{-1} using a Kurt J Lesker Thermal Evaporator (Nano 36). A shadow mask was used during thermal evaporation to define the device channel width (4500 µm) and channel length (70 µm). All device measurements were carried out in a nitrogen atmosphere inside a glove box using an Agilent Keysight semiconductor analyzer (B1500A) and a probe station at room temperature. The mobilities were calculated from the transfer curves using the equation $I_{DS} = (\mu W C_{eq}/2L)(V_{GS} - V_T)^2$, where I_{DS} represent the source–drain current, μ is the mobility, W and L are the channel width and length, C_{eq} is the capacitance per unit area of the substrate, and V_{GS} and V_T are the gate voltage and threshold voltage, respectively.

2. Synthesis of PDPPTT-Si$_n$

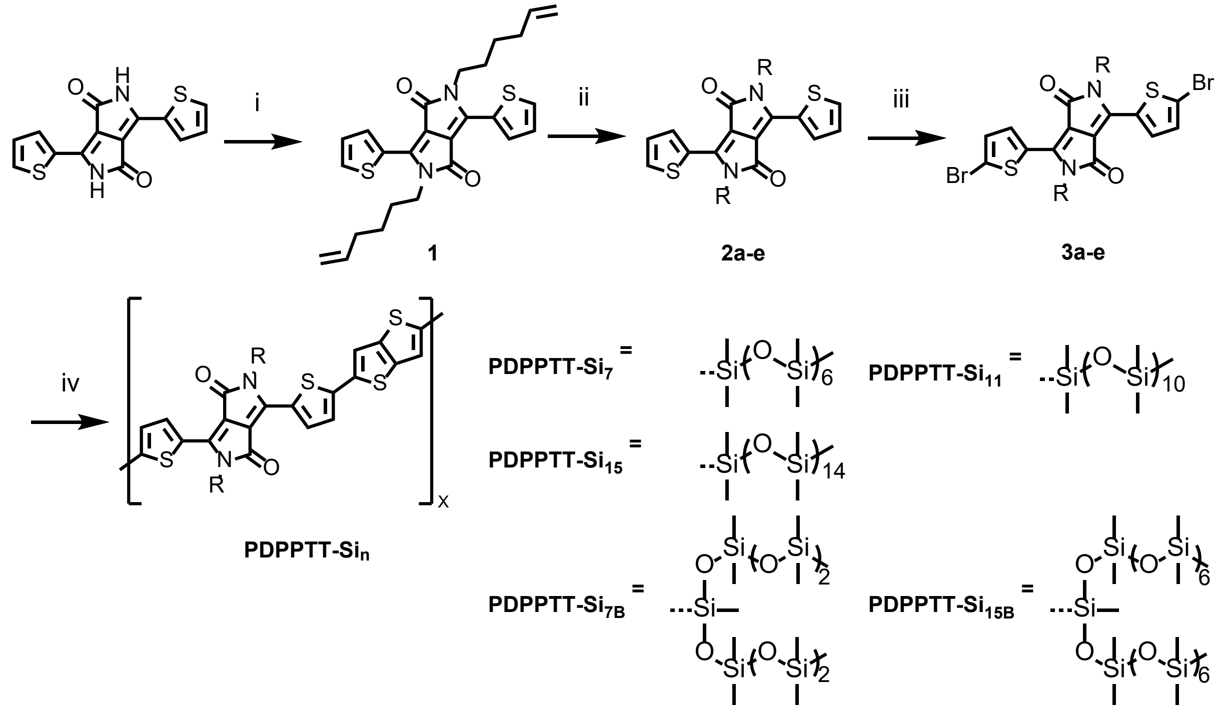

***Scheme S1**: Synthetic scheme towards **PDPPTT-Si$_n$**. (i) 6-bromo-1-hexene, K_2CO_3, 18-crown-6, DMF, 130 °C (49%). (ii) **Si$_n$H** , Karstedt's catalyst, toluene, 80 °C (37 – 67 %). (iii) NBS, $CHCl_3$, 0 °C (69 – 84 %). (iv) 2,5-bis(trimethylstannyl)-thieno[3,2-B]thiophene, $Pd(dba)_3$, P(o-tolyl)$_3$, toluene, 115 °C (60 – 82 %).*

2,5-di(hex-5-en-1-yl)-3,6-di(thiophen-2-yl)-2,5-dihydropyrrolo[3,4-c]pyrrole-1,4-dione (**1**).

A 250 mL round bottom flask was charged with 3,6-di(thiophen-2-yl)-2,5-dihydropyrrolo[3,4-c]pyrrole-1,4-dione (3.0 g, 10 mmol, 1 eq), K_2CO_3 (6.9 g, 50 mmol, 5 eq), 18-crown-6 (0.61 g, 2.3, 0.2 eq), in dry DMF (90 mL). The reaction mixture was stirred at 100 °C for two hours, after which 6-bromo-1-hexene (7.5 mL, 56 mmol, 5.6 eq) was added dropwise. The reaction mixture was stirred at 110 °C for 72 hours. DMF was removed under reduced pressure and the crude material was dissolved in chloroform and washed with water (3x 200 mL). The organic layer was collected and dried over $MgSO_4$. The crude material was further purified using automated silica gel column chromatography (Reveleris Silica Flash Cartridge 120 g, eluent cyclohexane/DCM 20/80-60/40, solid loading onto celite, elution after 10 CV @ 80/20), yielding a dark red solid after being dried in the vacuum oven overnight (2.3 g, 49%).

^{1}H NMR (400 MHz, Chloroform-*d*) δ 8.92 (dd, J = 3.9, 1.2 Hz, 2H, C-C-S-C$\underline{H}$CHCH), 7.64 (dd, J = 5.0, 1.2 Hz, 2H, C-C-S-CHCHC$\underline{H}$), 7.29 (dd, J = 5.1, 3.9 Hz, 2H, C-C-S-CHC$\underline{H}$CH), 5.79 (dd, J = 16.9, 10.2, 6.6 Hz, 2H, $CH_2C\underline{H}CH_2CH_2CH_2CH_2N$), 5.04 – 4.92 (m, 4H, $C\underline{H}_2CHCH_2CH_2CH_2CH_2N$), 4.12 – 4.06 (m, 4H, $CH_2CHCH_2CH_2CH_2C\underline{H}_2N$), 2.15 – 2.07 (m, 4H, $CH_2CHC\underline{H}_2CH_2CH_2CH_2N$), 1.82 – 1.72 (m, 4H, $CH_2CHCH_2CH_2C\underline{H}_2CH_2N$), 1.57 – 1.48 (m, 4H, $CH_2CHCH_2C\underline{H}_2CH_2CH_2N$). ^{13}C NMR (100 MHz, Chloroform-d) δ 161.37, 140.00, 138.29,

135.28, 130.70, 129.72, 128.65, 114.90, 107.70, 42.03, 33.34, 29.48. MALDI-TOF MS m/z calcd for $[M+H]^+$ 464.16, obsd 464.16; calcd for $[M+Na]^+$ 487.15, obsd 487.14. FT-IR *v* (cm^{-1}) = 3086, 3065, 2927, 2855, 1655, 1562, 1460, 1403, 1204, 1105, 1060, 910, 833, 733, 699.

General method A for hydrosilylations

A 250 mL round bottom flask was charged with **1** (1 mmol, 1 eq) and the corresponding discrete siloxane (4 eq) in dry toluene (50 mL). When all components were dissolved, 1-2 drop(s) of Karstedt catalyst in xylene solution (2 wt%) were added. The reaction was stirred at 80 °C for 4-16 hours. The solvent was removed under reduced pressure. The crude was dissolved in diethyl ether and washed with water (3x 150 mL). The organic layer was collected and dried over $MgSO_4$. The crude material was further purified using automated silica gel column chromatography (Reveleris Silica Flash Cartridge 220 g, eluent cyclohexane/DCM 20/80-50/50, solid loading onto celite, elution after 5 CV @ 50/50), yielding a dark purple oil after being dried in the vacuum oven overnight.

*2,5-bis(5-(1,1,3,3,5,5,7,7,9,9,11,11,13,13,13-pentadecamethylheptasiloxaneyl)pentyl)-3,6-di(thiophen-2-yl)-2,5-dihydropyrrolo[3,4-c]pyrrole-1,4-dione (**2a**).*

The synthesis of **2a** was performed following general method A, yielding a dark purple oil (860.6 mg, 53%).

^{1}H NMR (400 MHz, Chloroform-*d*) δ 8.93 (dd, J = 3.9, 1.2 Hz, 2H, C-C-S-C$\underline{H}$CHCH), 7.63 (dd, J = 5.0, 1.2 Hz, 2H, C-C-S-CHCHC$\underline{H}$), 7.28 (dd, J = 5.1, 3.9 Hz, 2H, C-C-S-CHC$\underline{H}$CH), 4.10 – 4.04 (m, 4H, $CH_2CH_2CH_2CH_2CH_2C\underline{H}_2N$), 1.74 (p, J = 8.3 Hz, 4H, $CH_2CH_2CH_2CH_2C\underline{H}_2CH_2N$), 1.46 – 1.29 (m, 12H, $CH_2C\underline{H}_2C\underline{H}_2C\underline{H}_2CH_2CH_2N$), 0.53 (dd, J = 10.3, 5.7 Hz, 4H, $C\underline{H}_2$-*o*DMS), 0.09 – 0.03 (m, 90H, *o*DMS). ^{13}C NMR (100 MHz, Chloroform-*d*) δ 160.29, 138.93, 134.18, 129.56, 128.71, 127.53, 106.62, 41.17, 31.97, 28.87, 25.57, 22.11, 17.13, -1.08. MALDI-TOF MS m/z calcd for $[M+H]^+$ 1500.50, obsd 1500.51; calcd for $[M+Na]^+$ 1523.49, obsd 1525.53. FT-IR *v* (cm^{-1}) = 2960, 2927, 2858, 1667, 1565, 1400, 1256, 1018, 787, 699.

*3,6-di(thiophen-2-yl)-2,5-bis(6-(1,1,3,3,5,5,7,7,9,9,11,11,13,13,15,15,17,17,19,19,21,21,21-tricosamethylundecasiloxaneyl)hexyl)-2,5-dihydropyrrolo[3,4-c]pyrrole-1,4-dione (**2b**).*

The synthesis of **2b** was performed following general method A, yielding a dark purple oil (277.3 mg, 60%).

^{1}H NMR (400 MHz, Chloroform-*d*) δ 8.93 (dd, J = 3.9, 1.2 Hz, 2H, C-C-S-C$\underline{H}$CHCH), 7.63 (dd, J = 5.0, 1.2 Hz, 2H, C-C-S-CHCHC$\underline{H}$), 7.28 (dd, J = 5.1, 3.9 Hz, 2H, C-C-S-CHC$\underline{H}$CH), 4.10 –

4.04 (m, 4H, $CH_2CH_2CH_2CH_2CH_2C\underline{H}_2N$), 1.79 – 1.69 (m, 4H, $CH_2CH_2CH_2CH_2C\underline{H}_2CH_2N$), 1.45 – 1.30 (m, 12H, $CH_2C\underline{H}_2C\underline{H}_2C\underline{H}_2CH_2CH_2N$), 0.53 (dd, *J* = 10.0, 5.9 Hz, 4H, $C\underline{H}_2$-*o*DMS), 0.13 – 0.02 (m, 138H, *o*DMS). ^{13}C NMR (100 MHz, Chloroform-*d*) δ 160.31, 134.20, 129.57, 128.72, 127.55, 41.19, 31.99, 28.89, 25.59, 22.13, 17.14, -1.07. MALDI-TOF-MS m/z calcd for $[M+H]^+$ 2092.65, obsd 2095.66; calcd for $[M+Na]^+$ 2115.64, obsd 2117.66; calcd for $[M+K]^+$ 2131.75, obsd 2134.64; calcd for $[M-CH_3+H]^+$ 2077.65, obsd 2082.64; calcd for $[M-2CH_3+H]^+$ 2065.65, obsd 2070.65. FT-IR *v* (cm^{-1}) = 2964, 1668, 1564, 1403, 1257, 1080, 1013, 842, 787, 736, 702.

*2,5-bis(6-(1,1,3,3,5,5,7,7,9,9,11,11,13,13,15,15,17,17,19,19,21,21,23,23,25,25,27,27,29,29,29-hentriacontamethylpentadecasiloxaneyl)hexyl)-3,6-di(thiophen-2-yl)-2,5-dihydropyrrolo[3,4-c]pyrrole-1,4-dione (**2c**).*

The synthesis of **2c** was performed following general method A, yielding a dark purple oil (349.3 mg, 67%).

^{1}H NMR (400 MHz, Chloroform-*d*) δ 8.93 (dd, *J* = 3.9, 1.2 Hz, 2H, C-C-S-C$\underline{H}$CHCH), 7.63 (dd, *J* = 5.0, 1.2 Hz, 2H, C-C-S-CHCHC$\underline{H}$), 7.28 (dd, *J* = 5.0, 3.9 Hz, 2H, C-C-S-CHC$\underline{H}$CH), 4.13 – 4.02 (m, 4H, $CH_2CH_2CH_2CH_2CH_2C\underline{H}_2N$), 1.73 (q, *J* = 7.9, 7.0 Hz, 4H, $CH_2CH_2CH_2CH_2C\underline{H}_2CH_2N$), 1.46 – 1.25 (m, 12H, $CH_2C\underline{H}_2C\underline{H}_2C\underline{H}_2CH_2CH_2N$), 0.53 (dd, *J* = 10.2, 5.8 Hz, 4H, $C\underline{H}_2$-*o*DMS), 0.10 – 0.02 (m, 196H, *o*DMS). ^{13}C NMR (100 MHz, Chloroform-*d*) δ 160.32, 134.21, 129.58, 128.74, 127.55, 41.20, 32.00, 28.90, 25.60, 22.14, 17.15, -1.06. MALDI-TOF-MS m/z calcd for $[M+H]^+$ 2684.80, obsd 2688.81; calcd for $[M+Na]^+$ 2707.79, obsd 2711.80; calcd for $[M+K]^+$ 2723.9, obsd 2725.80; calcd for $[M-CH_3+H]$ 2669.8, obsd 2678.79. FT-IR *v* (cm^{-1}) = 2961, 1665, 1562, 1406, 1261, 1010, 789, 701.

*2,5-bis(6-(1,1,1,3,3,5,5,7,9,9,11,11,13,13,13-pentadecamethylheptasiloxan-7-yl)hexyl)-3,6-di(thiophen-2-yl)-2,5-dihydropyrrolo[3,4-c]pyrrole-1,4-dione (**2d**).*

The synthesis of **2d** was performed following general method A, yielding a dark purple oil (221.7 mg, 59%).

^{1}H NMR (400 MHz, Chloroform-*d*) δ 8.93 (dd, *J* = 3.9, 1.1 Hz, 2H, C-C-S-C$\underline{H}$CHCH), 7.63 (dd, *J* = 5.0, 1.1 Hz, 2H, C-C-S-CHCHC$\underline{H}$), 7.28 (dd, *J* = 5.0, 3.9 Hz, 2H, C-C-S-CHC$\underline{H}$CH), 4.11 – 4.02 (m, 4H, $CH_2CH_2CH_2CH_2CH_2C\underline{H}_2N$), 1.79 – 1.70 (m, 4H, $CH_2CH_2CH_2CH_2C\underline{H}_2CH_2N$), 1.47 – 1.31 (m, 12H, $CH_2C\underline{H}_2C\underline{H}_2C\underline{H}_2CH_2CH_2N$), 0.50 (t, *J* = 7.8 Hz, 4H, $C\underline{H}_2$-*o*DMS), 0.07 (d, *J* =

8.1 Hz, 96H, *o*DMS). ^{13}C NMR (100 MHz, Chloroform-*d*) *δ* 159.54, 138.18, 133.45, 128.80, 127.97, 126.79, 105.88, 40.44, 31.19, 28.20, 24.89, 21.19, 15.66, -1.82, -2.30. MALDI-TOF-MS m/z calcd for $[M+H]^+$ 1500.50, obsd 1502.50; calcd for $[M+Na]^+$ 1523.49, obsd 1525.50. FT-IR *v* (cm^{-1}) = 2957, 1665, 1562, 1402, 1254, 1021, 785, 697.

*2,5-bis(6-(1,1,1,3,3,5,5,7,7,9,9,11,11,13,13,15,17,17,19,19,21,21,23,23,25,25,27,27,29,29,29-hentriacontamethylpentadecasiloxan-15-yl)hexyl)-3,6-di(thiophen-2-yl)-2,5-dihydropyrrolo[3,4-c]pyrrole-1,4-dione (**2e**).*

The synthesis of **2e** was performed following general method A, yielding a dark purple oil (221.0 mg, 37%).

^1H NMR (400 MHz, Chloroform-*d*) *δ* 8.93 (dd, *J* = 4.0, 1.2 Hz, 2H, C-C-S-C$\underline{H}$CHCH), 7.62 (dd, *J* = 5.0, 1.2 Hz, 2H, C-C-S-CHCHC$\underline{H}$), 7.28 (dd, *J* = 5.0, 3.9 Hz, 2H, C-C-S-CHC$\underline{H}$CH), 4.09 – 4.03 (m, 4H, $CH_2CH_2CH_2CH_2CH_2C\underline{H}_2N$), 1.78 – 1.70 (m, 4H, $CH_2CH_2CH_2CH_2C\underline{H}_2CH_2N$), 1.39 (dt, *J* = 21.1, 5.9 Hz, 12H, $CH_2C\underline{H}_2C\underline{H}_2C\underline{H}_2CH_2CH_2N$), 0.50 (t, *J* = 7.8 Hz, 4H, $C\underline{H}_2$-*o*DMS)), 0.10 – 0.02 (m, 186H, *o*DMS). MALDI-TOF-MS m/z calcd for $[M+H]^+$ 2684.80, obsd 2688.81; calcd for $[M+Na]^+$ 2707.79, obsd 2712.81; calcd for $[M+K]^+$ 2723.90, obsd 2723.82; calcd for $[M-CH_3+H]$ 2669.80, obsd 2647.78. FT-IR *v* (cm^{-1}) = 2957, 1675, 1556, 1406, 1258, 1009, 783, 686.

General method B for brominations

A 250 mL round bottom flask was covered in aluminum foil and charged with **2** (0.80 mg, 0.53 mmol, 1 eq) in 100 mL chloroform. The flask was cooled to 0 °C under argon, after which N-bromo succinimide (0.21 g, 1.2 mmol, 2.2 eq) was added portion-wise. The solution was stirred for 24h. The completion of the reaction was assessed with TLC. If TLC showed less than full conversion the reaction mixture was stirred for another night. The reaction mixture was poured into water to quench the reaction. DCM was added to the mixture and was extracted with water (3x 150 mL). The organic layer was collected and dried over $MgSO_4$. The crude material was further purified using automated silica gel column chromatography (Reveleris Silica Flash Cartridge 80 g, eluent cyclohexane/DCM 30/70-50/50, solid loading onto celite, elution after 3 CV @ 50/50), yielding a dark purple solid after being dried in the vacuum oven overnight.

*3,6-bis(5-bromothiophen-2-yl)-2,5-bis(6-(1,1,3,3,5,5,7,7,9,9,11,11,13,13,13-pentadecamethylheptasiloxaneyl)hexyl)-2,5-dihydropyrrolo[3,4-c]pyrrole-1,4-dione (**3a**).*

The synthesis of **3a** was performed following general method B using **2a**, yielding a dark purple solid (735.0 mg, 84%).

^{1}H NMR (400 MHz, Chloroform-*d*) δ 8.68 (d, J = 4.2 Hz, 2H, C-C-S-CBr-CHC$\underline{H}$), 7.24 (d, J = 4.2 Hz, 2H, C-C-S-CBr-C$\underline{H}$CH), 4.01 – 3.95 (m, 4H, $CH_2CH_2CH_2CH_2CH_2C\underline{H}_2N$), 1.67 (s, 4H, $CH_2CH_2CH_2CH_2C\underline{H}_2CH_2N$), 1.44 – 1.30 (m, 12H, $CH_2C\underline{H}_2C\underline{H}_2C\underline{H}_2CH_2CH_2N$), 0.53 (dd, J = 9.8, 6.1 Hz, 4H, $C\underline{H}_2$-*o*DMS), 0.12 – 0.02 (m, 90H, *o*DMS). ^{13}C NMR (100 MHz, Chloroform-*d*) δ 159.95, 137.90, 134.28, 130.57, 130.03, 118.05, 106.72, 41.23, 31.92, 28.91, 25.52, 22.08, 17.11, -1.09. MALDI-TOF-MS m/z calcd for $[M+H]^+$ 1656.32, obsd 1660.36; calcd for $[M+Na]^+$ 1679.31, obsd 1683.35. FT-IR v (cm^{-1}) = 2961, 1654, 1558, 1417, 1257, 1395, 1021, 785, 720.

*3,6-bis(5-bromothiophen-2-yl)-2,5-bis(6-(1,1,3,3,5,5,7,7,9,9,11,11,13,13,15,15,17,17,19,19,21,21,21-tricosamethylundecasiloxaneyl)hexyl)-2,5-dihydropyrrolo[3,4-c]pyrrole-1,4-dione (**3b**).*

The synthesis of **3b** was performed following general method B using **2b** (with a reaction time of 72h)**,** yielding a dark purple solid (179.2 mg, 75%).

^{1}H NMR (400 MHz, Chloroform-*d*) δ 8.68 (d, J = 4.3 Hz, 2H, C-C-S-CBr-CHC$\underline{H}$), 7.24 (d, J = 4.2 Hz, 2H, C-C-S-CBr-C$\underline{H}$CH), 3.02 – 3.94 (m, 4H, $CH_2CH_2CH_2CH_2CH_2C\underline{H}_2N$), 1.70 (d, J = 7.6 Hz, 4H, $CH_2CH_2CH_2CH_2C\underline{H}_2CH_2N$), 1.38 (m, 12H, $CH_2C\underline{H}_2C\underline{H}_2C\underline{H}_2CH_2CH_2N$), 0.53 (dd, J = 9.9, 5.9 Hz, 4H, $C\underline{H}_2$-*o*DMS), 0.12-0.02 (dt, J = 7.3, 5.3 Hz, 138H, *o*DMS). ^{13}C NMR (100 MHz, Chloroform-*d*) δ 159.97, 134.29, 130.59, 130.05, 118.07, 106.74, 41.25, 31.94, 28.93, 25.54, 22.10, 17.13, -1.07. MALDI-TOF-MS m/z calcd for $[M+H]^+$ 2248.47, obsd 2253.42; calcd for $[M+Na]^+$ 2271.46, obsd 2276.52; calcd for $[M+K]^+$ 2287.57, obsd 2292.49; calcd for $[M-CH_3+H]$ 2233.47, obsd 2239.50; calcd for $[M-2CH_3+H]^+$ 2218.47, obsd 2229.51. FT-IR v (cm^{-1}) = 2961, 1657, 1558, 1414, 1395, 1257, 1014, 785, 724, 697.

*3,6-bis(5-bromothiophen-2-yl)-2,5-bis(6-(1,1,3,3,5,5,7,7,9,9,11,11,13,13,15,15,17,17,19,19,21,21,23,23,25,25,27,27,29,29,29-hentriacontamethylpentadecasiloxaneyl)hexyl)-2,5-dihydropyrrolo[3,4-c]pyrrole-1,4-dione (**3c**).*

The synthesis of **3c** was performed following general method B using **2c** (with a reaction time of 72h), yielding a dark purple solid (228.6 mg, 71%).

^{1}H NMR (400 MHz, Chloroform-*d*) δ 8.68 (d, *J* = 4.2 Hz, 2H, C-C-S-CBr-CHC<u>H</u>), 7.24 (d, *J* = 4.2 Hz, 2H, C-C-S-CBr-C<u>H</u>CH), 4.01 – 3.95 (m, 4H, $CH_2CH_2CH_2CH_2CH_2C\underline{H}_2N$), 1.75 – 1.67 (m, 4H, $CH_2CH_2CH_2CH_2C\underline{H}_2CH_2N$) , 1.37 (dt, *J* = 15.3, 5.0 Hz, 12H, $CH_2C\underline{H}_2C\underline{H}_2C\underline{H}_2CH_2CH_2N$), 0.56 – 0.50 (m, 4H, $C\underline{H}_2$-*o*DMS), 0.13 – 0.02 (m, 195H, *o*DMS). ^{13}C NMR (100 MHz, Chloroform-*d*) δ 159.98, 134.31, 130.60, 130.06, 41.26, 31.95, 28.94, 25.55, 22.11, 17.14, -1.06. MALDI-TOF-MS m/z calcd for $[M+H]^+$ 2840.62, obsd 2846.70; calcd for $[M+Na]^+$ 2863.61, obsd 2869.69; calcd for $[M+K]^+$ 2879.72, obsd 2885.67; calcd for $[M-CH_3+H]$ 2825.62, obsd 2831.68. FT-IR *v* (cm^{-1}) = 2961, 1661, 1558, 1417, 1254, 1010, 781.

*3,6-bis(5-bromothiophen-2-yl)-2,5-bis(6-(1,1,1,3,3,5,5,7,9,9,11,11,13,13,13-pentadecamethylheptasiloxan-7-yl)hexyl)-2,5-dihydropyrrolo[3,4-c]pyrrole-1,4-dione (**3d**).*

The synthesis of **3d** was performed following general method B using **2d**, yielding a dark sticky solid (173.6 mg, 80%).

^{1}H NMR (400 MHz, Chloroform-*d*) δ 8.68 (d, *J* = 4.2 Hz, 2H, C-C-S-CBr-CHC<u>H</u>), 7.23 (d, *J* = 4.2 Hz, 2H, C-C-S-CBr-C<u>H</u>CH), 4.01 – 3.95 (m, 4H, $CH_2CH_2CH_2CH_2CH_2C\underline{H}_2N$), 1.75 – 1.66 (m, 4H, $CH_2CH_2CH_2CH_2C\underline{H}_2CH_2N$), 1.43 – 1.33 (m, 12H, $CH_2C\underline{H}_2C\underline{H}_2C\underline{H}_2CH_2CH_2N$), 0.51 (t, *J* = 7.7 Hz, 4H, $C\underline{H}_2$-*o*DMS), 0.07 (d, *J* = 7.9 Hz, 90H, *o*DMS). ^{13}C NMR (100 MHz, Chloroform-*d*) δ 159.19, 137.15, 133.53, 129.82, 129.29, 117.30, 105.98, 40.49, 31.17, 28.26, 24.87, 21.17, 15.65, -1.83, -2.29. MALDI-TOF-MS m/z calcd for $[M+H]^+$ 1656.32, obsd 1660.34; calcd for $[M+Na]^+$ 1683.33, obsd 1683.34; calcd for $[M+K]^+$ 1699.44, obsd 1699.31; calcd for $[M-CH_3+H]$ 1645.34, obsd 1647.33. FT-IR *v* (cm^{-1}) = 2957, 1667, 1555, 1402, 1254, 1017, 796, 678.

*3,6-bis(5-bromothiophen-2-yl)-2,5-bis(6-(1,1,1,3,3,5,5,7,7,9,9,11,11,13,13,15,17,17,19,19,21,21,23,23,25,25,27,27,29,29,29-hentriacontamethylpentadecasiloxan-15-yl)hexyl)-2,5-dihydropyrrolo[3,4-c]pyrrole-1,4-dione (**3e**).*

The synthesis of **3e** was performed following general method B using **2e** (with a reaction time of 48h), yielding a dark purple oil (132.0 mg, 69%).

^{1}H NMR (400 MHz, Chloroform-*d*) δ 8.68 (d, *J* = 4.2 Hz, 2H, C-C-S-CBr-CHC<u>H</u>), 7.23 (d, *J* = 4.2 Hz, 2H, C-C-S-CBr-C<u>H</u>CH), 4.01 – 3.94 (m, 4H, $CH_2CH_2CH_2CH_2CH_2C\underline{H}_2N$), 1.68 (d, *J* = 7.8 Hz, 4H, $CH_2CH_2CH_2CH_2C\underline{H}_2CH_2N$), 1.37 (s, 12H, $CH_2C\underline{H}_2C\underline{H}_2C\underline{H}_2CH_2CH_2N$), 0.50 (t, *J* = 7.7 Hz, 4H, $C\underline{H}_2$-*o*DMS), 0.09 – 0.06 (m, 172H, *o*DMS). ^{13}C NMR (100 MHz, Chloroform-*d*) δ

159.94, 137.90, 134.28, 130.58, 130.04, 118.05, 106.73, 41.26, 31.90, 29.01, 25.63, 21.94, 16.39, -1.07, -1.55. MALDI-TOF-MS m/z calcd for $[M+H]^+$ 2840.62, obsd 2846.69; calcd for $[M+Na]^+$ 2863.61, obsd 2869.68; calcd for $[M+K]^+$ 2879.72, obsd 2869.68; calcd for $[M-CH_3+H]$ 2825.62, obsd 2832.66. FT-IR v (cm^{-1}) = 2961, 1671, 1558, 1403, 1256, 1009, 783, 682.

General method C for polymerizations

A Schlenk flask was charged with **3** (0.30 g, 0.18 mmol, 1 eq), TT (84 mg, 0.18 mmol, 1 eq), catalyst $Pd_2(dba)_3$ (3.4 mg, 0.0037 mmol, 0.2 eq) and ligand P(*o*-tolyl)$_3$ (4.5 mg, 0.014 mmol, 0.08 eq) in dry toluene (44 mL). Oxygen was removed from the reaction mixture by repeating three freeze-pump-thaw cycles. The reaction mixture was subsequently heated to 115 °C and stirred for 24 hours. Thereafter, the product was precipitated in methanol. The precipitate was purified by Soxhlet extraction ethanol, acetone, cyclohexane, and chloroform to remove low molecular weight fractions. The resulting fractions were dried in the vacuum oven overnight, yielding a green solid.

PDPPTT-Si$_7$

The synthesis of ***PDPPTT-Si$_7$*** was performed following general method C using **3a**, yielding a green solid from the chloroform fraction (231.0 mg, 60%). GPC (*o*-DCB, 140 °C) M_n = 111 kDa, M_w = 347 kDa, PDI = 3.1. Due to tailing and aggregation these numbers are approximate.

PDPPTT-Si$_{11}$

The synthesis of ***PDPPTT-Si$_{11}$*** was performed following general method C using **3b**. A green solid was collected from the cyclohexane fraction (144.4 mg, 80%). GPC (*o*-DCB, 140 °C) M_n = 42 kDa, M_w = 141 kDa, PDI = 3.4. Due to tailing and aggregation these numbers are approximate.

PDPPTT-Si$_{15}$

The synthesis of ***PDPPTT-Si$_{15}$*** was performed following general method C using **3c**. A green solid was collected from the cyclohexane fraction (174.9 mg, 82%). GPC (*o*-DCB, 140 °C) M_n = 33 kDa, M_w = 119 kDa, PDI = 3.6. Due to tailing and aggregation these numbers are approximate.

PDPPTT-Si$_{7B}$

The synthesis of ***PDPPTT-Si$_{7B}$*** was performed following general method C using **3d**. A green solid was collected from the cyclohexane fraction (186.9 mg, 73%). GPC (*o*-DCB, 140 °C) M_n

= 69 kDa, M_w = 235 kDa, PDI = 3.4. Due to tailing and aggregation these numbers are approximate.

PDPPTT-Si$_{15B}$

The synthesis of ***PDPPTT-Si$_{15B}$*** was performed following general method C using **3e**. A green solid was collected from the cyclohexane fraction (102 mg, 72%). GPC (*o*-DCB, 140 °C) M_n = 55 kDa, M_w = 116, PDI = 2.3. Due to tailing and aggregation these numbers are approximate.

3. Recycle GPC traces of PDPPTT-Si$_n$

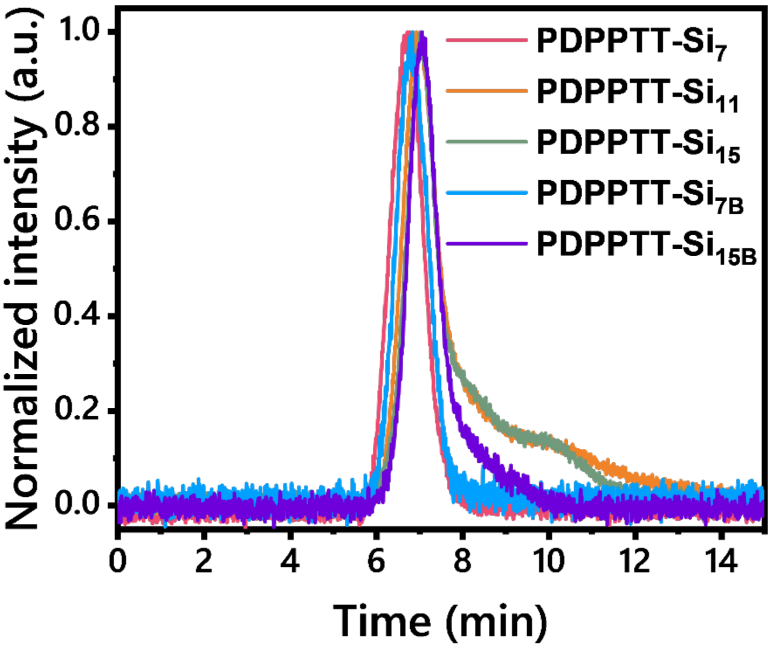

***Figure S1**: Gel permeation chromatograms of **PDPPTT-Si$_n$** in* o*-DCB at 140 ˚C. The tailing in the GPC trace of **PDPPTT-Si$_{11}$**, **PDPPTT-Si$_{15}$** and **PDPPTT-Si$_{15B}$** were excluded from the estimation of M_n and M_w.*

4. UV-Vis spectra of PDPPTT-Si_n in various solvents

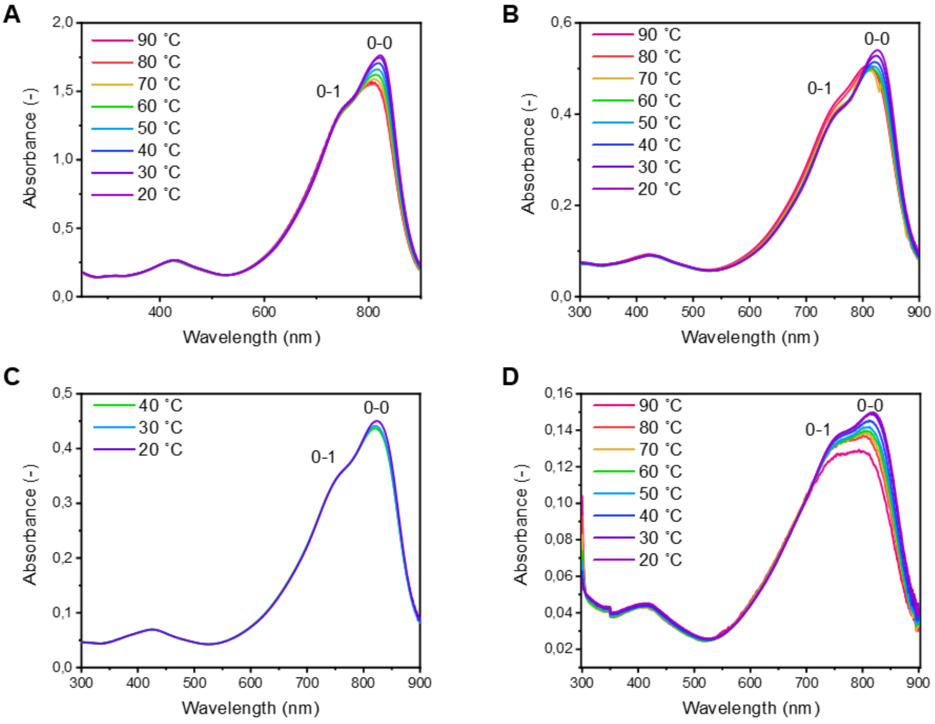

Figure S2: *Temperature-dependent UV-Vis spectra for* ***PDPPTT-Si₇*** *in a) MCH, b) toluene, c) CHCl₃ and d) o-DCB.*

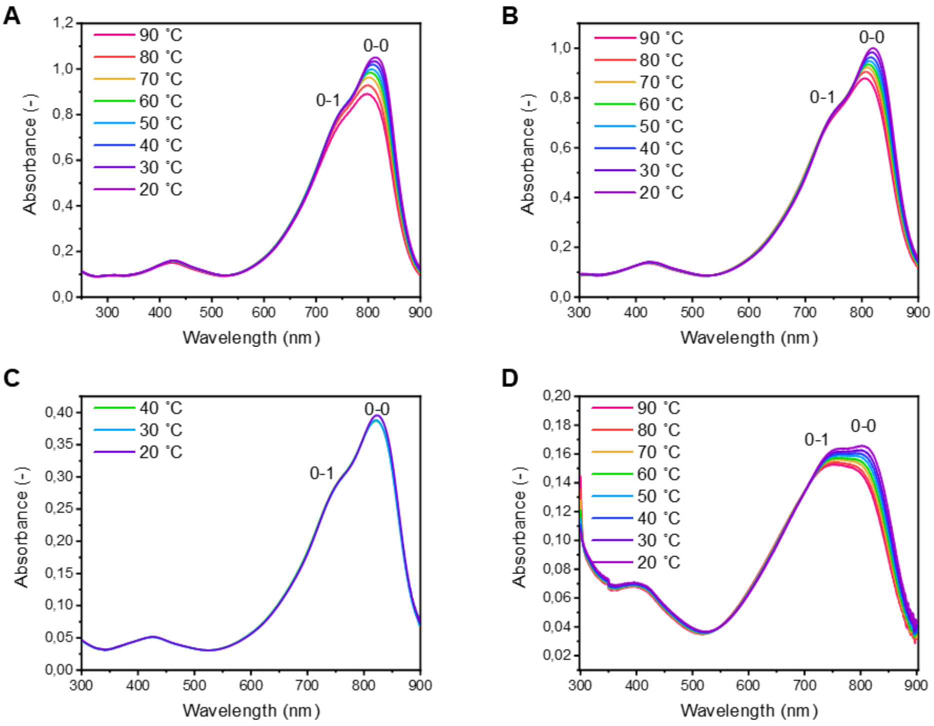

Figure S3: *Temperature-dependent UV-Vis spectra for* ***PDPPTT-Si₁₁*** *in a) MCH, b) toluene, c) CHCl₃ and d) o-DCB.*

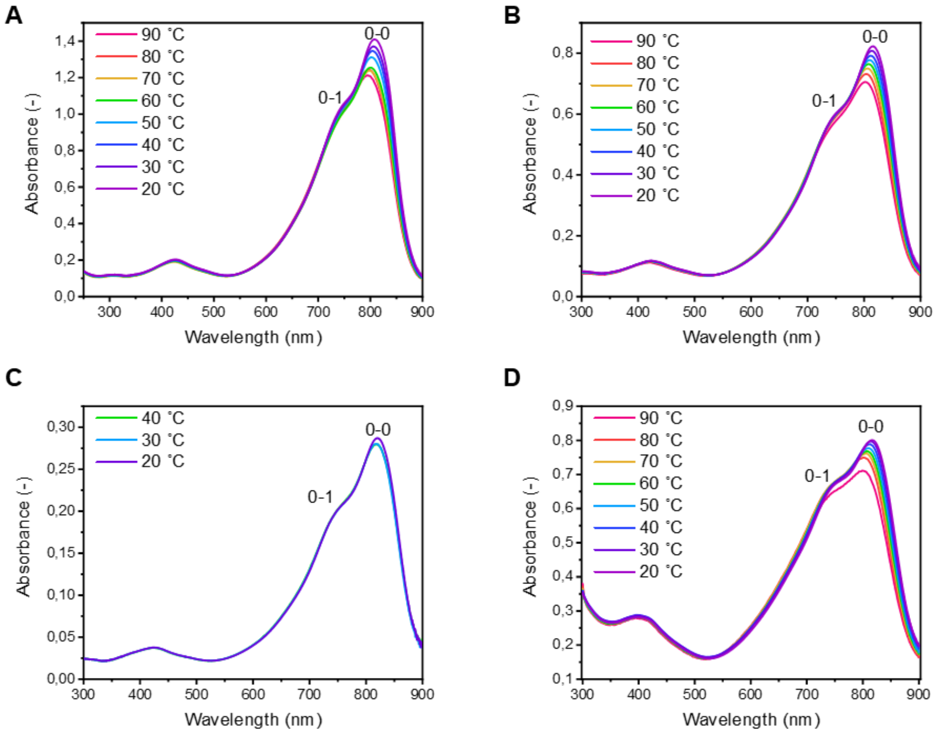

Figure S4: *Temperature-dependent UV-Vis spectra for* ***PDPPTT-Si$_{15}$*** *in a) MCH, b) toluene, c) $CHCl_3$ and d) o-DCB.*

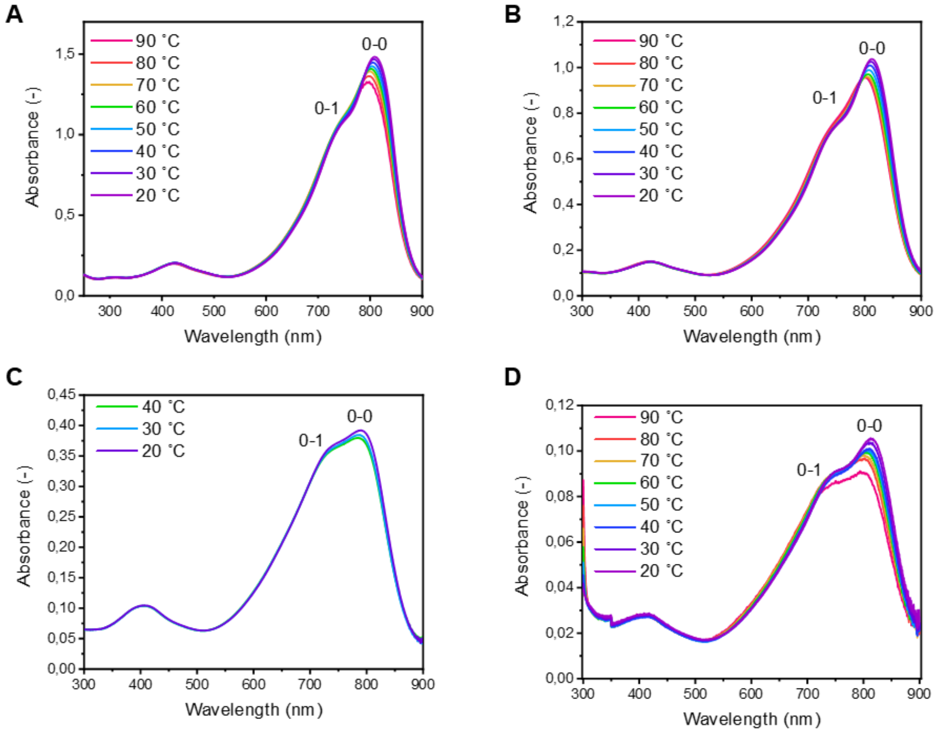

Figure S5: *Temperature-dependent UV-Vis spectra for* ***PDPPTT-Si$_{7B}$*** *in a) MCH, b) toluene, c) $CHCl_3$ and d) o-DCB.*

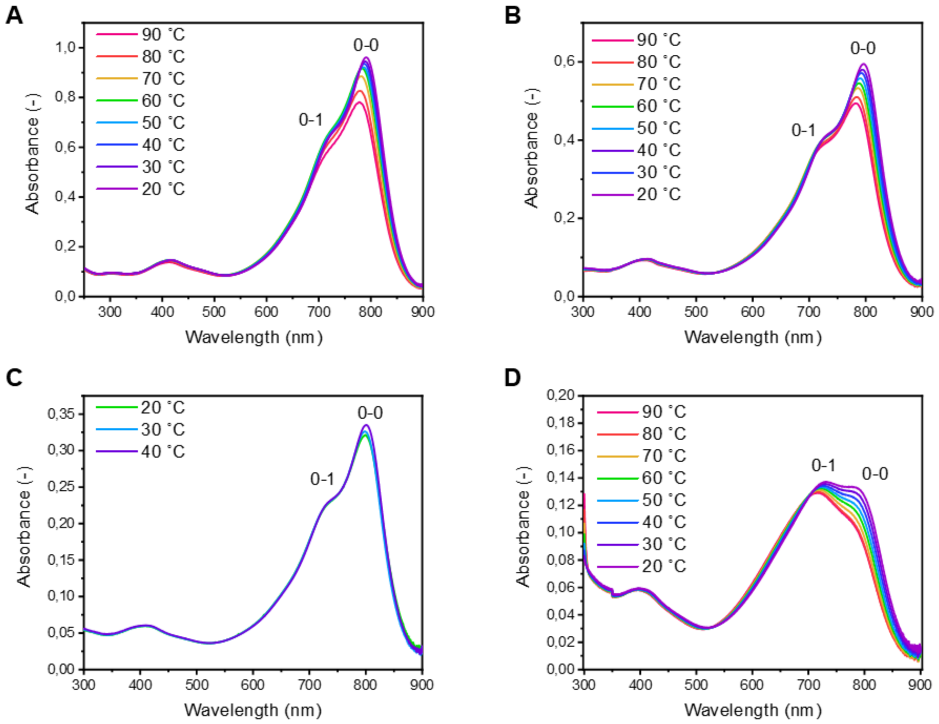

Figure S6: *Temperature-dependent UV-Vis spectra for **PDPPTT-Si$_{15B}$** in a) MCH, b) toluene, c) $CHCl_3$ and d) o-DCB.*

5. AFM imaging of PDPPTT-Si_{11}

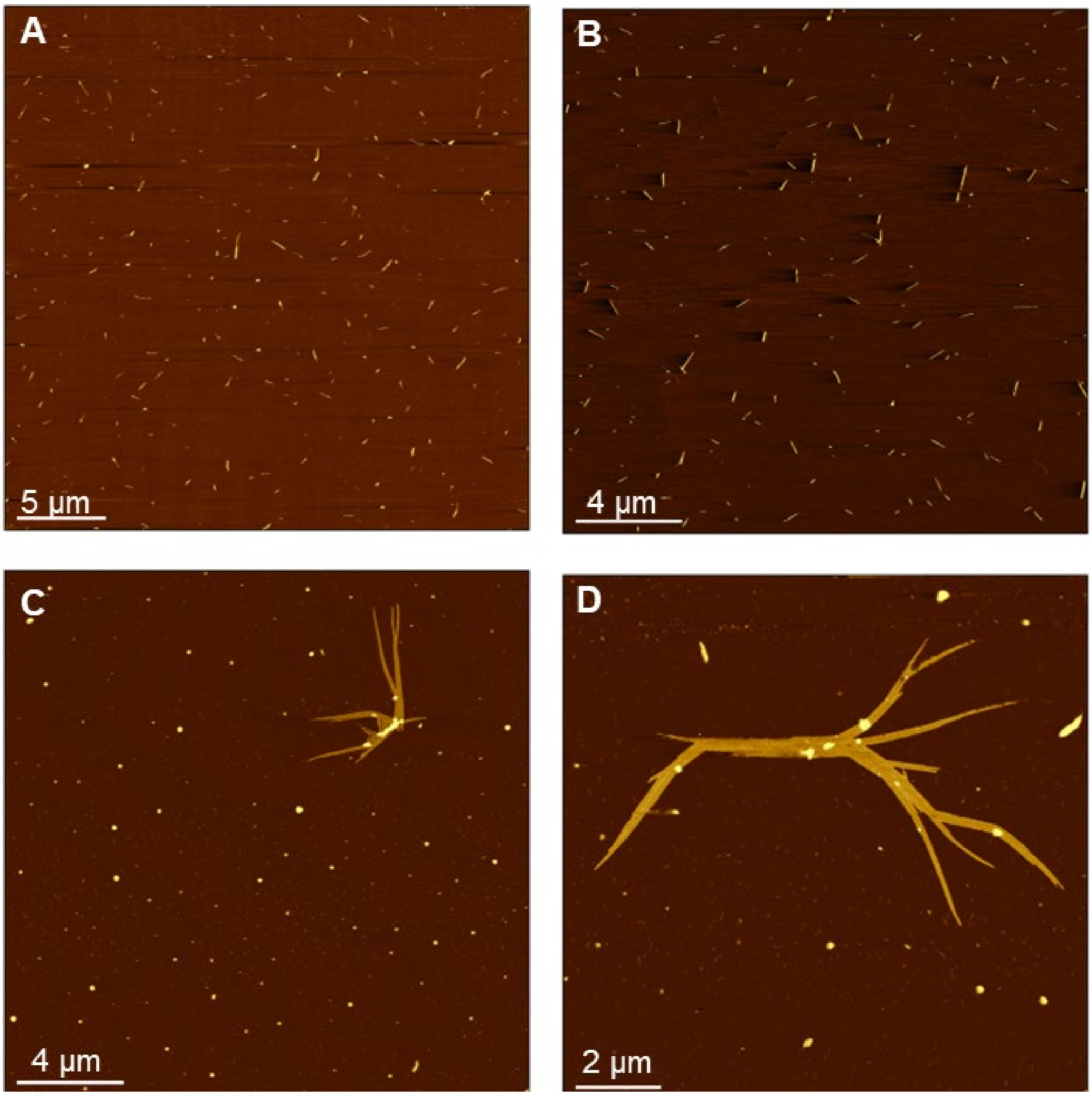

Figure S7: *Tapping mode AFM height images of spin coated solutions of* ***PDPPTT-Si_{11}*** *on freshly cleaved mica, where the light parts are the high regions, and the dark parts are the low regions. a) $8{\cdot}10^{-3}$ mg mL^{-1}, b) $8{\cdot}10^{-4}$ mg mL^{-1}, c, d) $8{\cdot}10^{-2}$ mg mL^{-1}.*

6. POM images of PDPPTT-Si_n

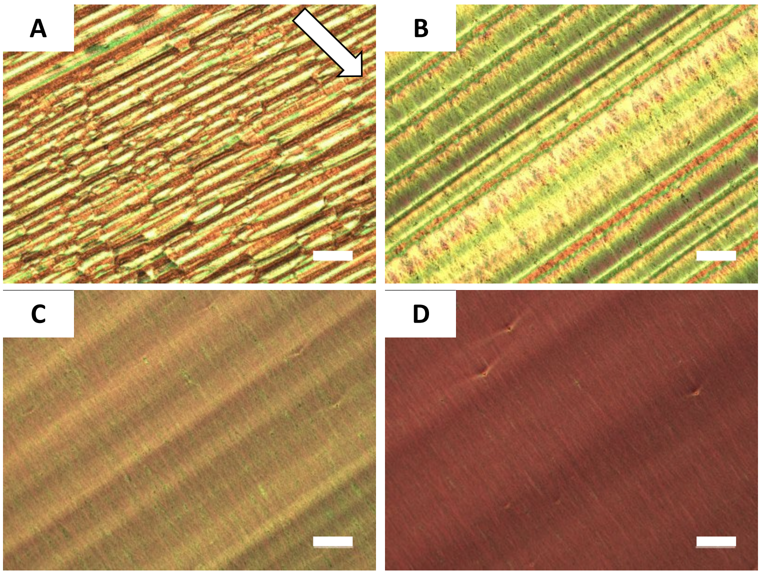

Figure S8: *Polarized microscope image of printed* ***PDPPTT-Si₇****, where the arrow represents the printing direction and the scale bar represents 100 µm. The printing speed was 0.1 mm/s (A), 0.3 mm/s (B), 0.5 mm/s (C) and 0.7 mm/s (D).*

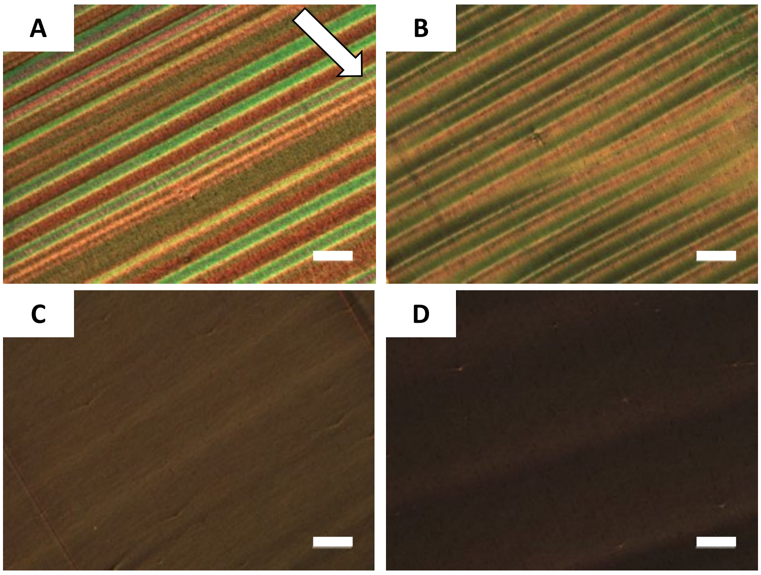

Figure S9*: Polarized microscope image of printed* ***PDPPTT-Si₁₁****, where the arrow represents the printing direction and the scale bar represents 100 µm. The printing speed was 0.1 mm/s (A), 0.3 mm/s (B), 0.5 mm/s (C) and 0.7 mm/s (D).*

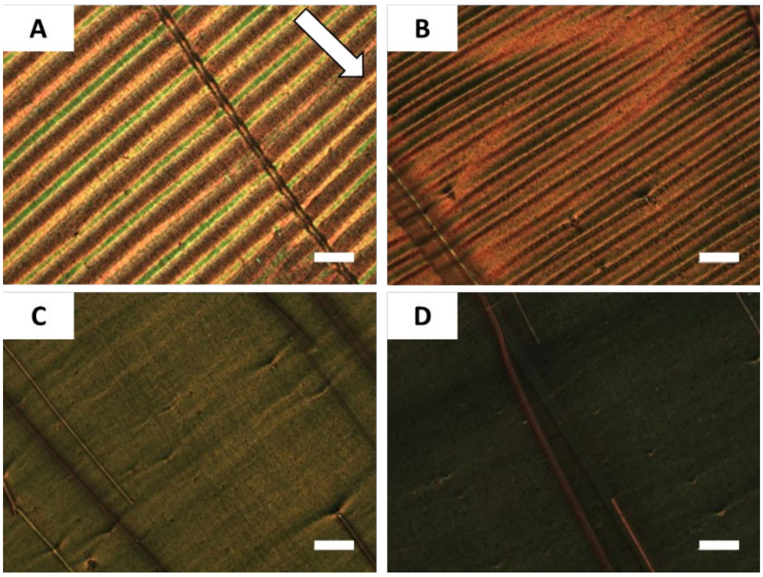

Figure S10: *Polarized microscope image of printed* ***PDPPTT-Si$_{15}$****, where the arrow represents the printing direction and the scale bar represents 100 µm. The printing speed was 0.1 mm/s (A), 0.3 mm/s (B), 0.5 mm/s (C) and 0.7 mm/s (D).*

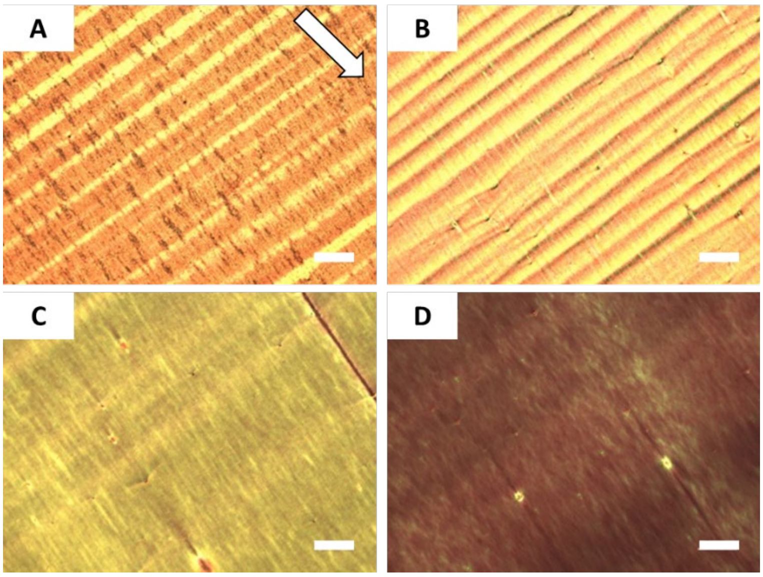

Figure S11: *Polarized microscope image of printed* ***PDPPTT-Si$_{7B}$****, where the arrow represents the printing direction and the scale bar represents 100 µm. The printing speed was 0.1 mm/s (A), 0.3 mm/s (B), 0.5 mm/s (C) and 0.7 mm/s (D).*

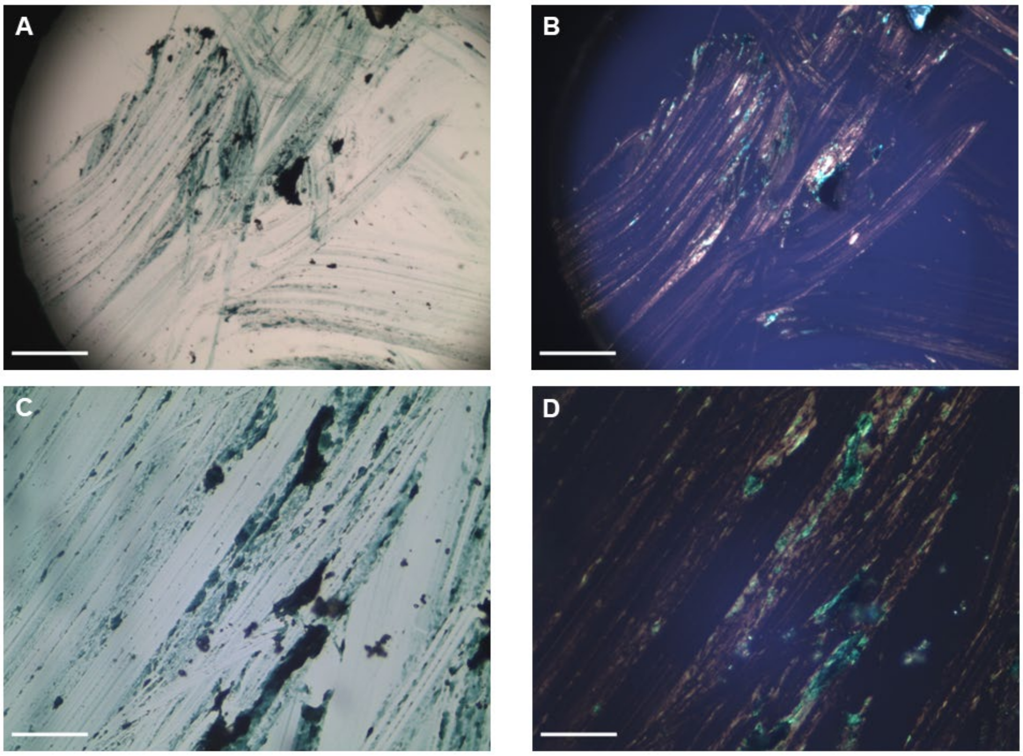

Figure S12: *a) Unpolarized and b) polarized microscope image of smeared* ***PDPPTT-Si$_{15B}$****, as no OFET device was fabricated from this material. The scale bar represents 400 µm. c) Unpolarized and d) polarized microscope image of* ***PDPPTT-Si$_{15B}$****, where the scale bar represents 65 µm.*

7. Thermal and Mechanical properties of PDPPTT-Si_n

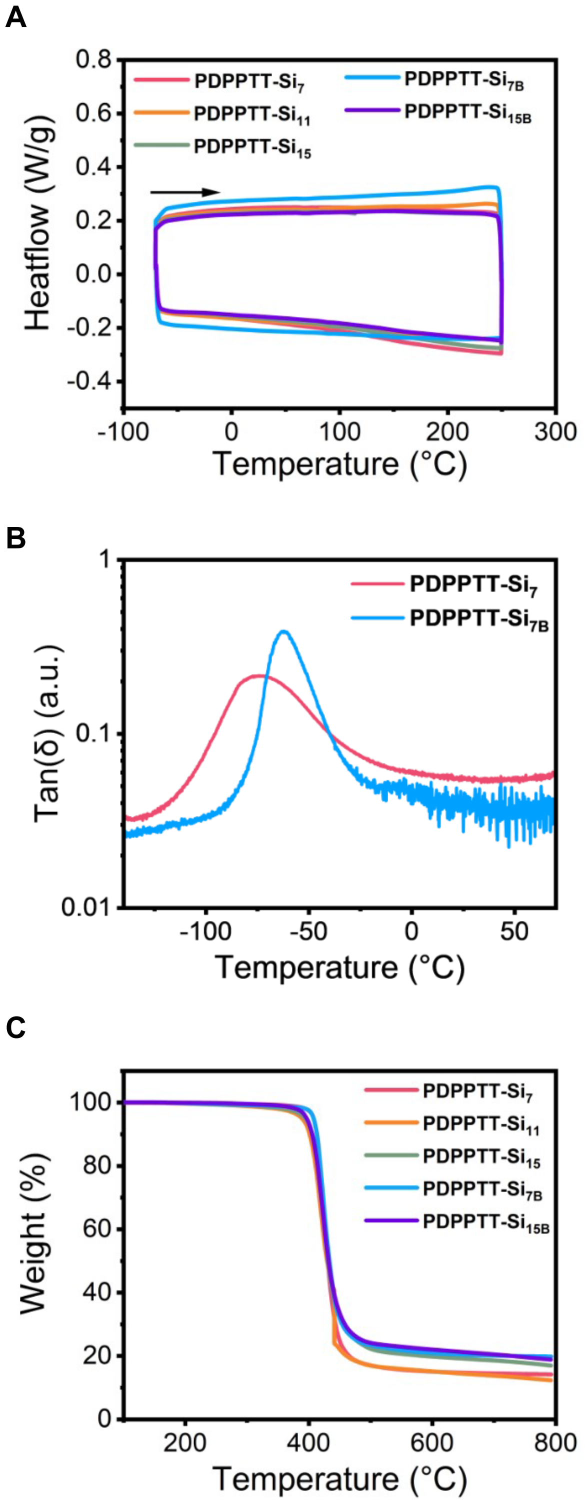

Figure S13: *(A) DSC thermogram of **PDPPTT-Si_n**. Endothermic transitions are shown up, the arrow indicates the direction of the heating/cooling cycle (rate = 10 °C min^{-1}). For all samples, the second cycle is displayed. (B) Tan(δ) of **PDPPTT-Si_7** and **PDPPTT-Si_{7B}** as a function of temperature. A heating rate of 3 °C min^{-1} at a frequency of 1 Hz, and strain of 0.1 % were applied. (C) TGA curves of the polymers with a heating rate of 10 °C min^{-1} under N_2.DMA traces of **PDPPTT-Si_7** and **PDPPTT-Si_{7B}**.*

8. Square wave voltammograms of PDPPTT-Si$_n$

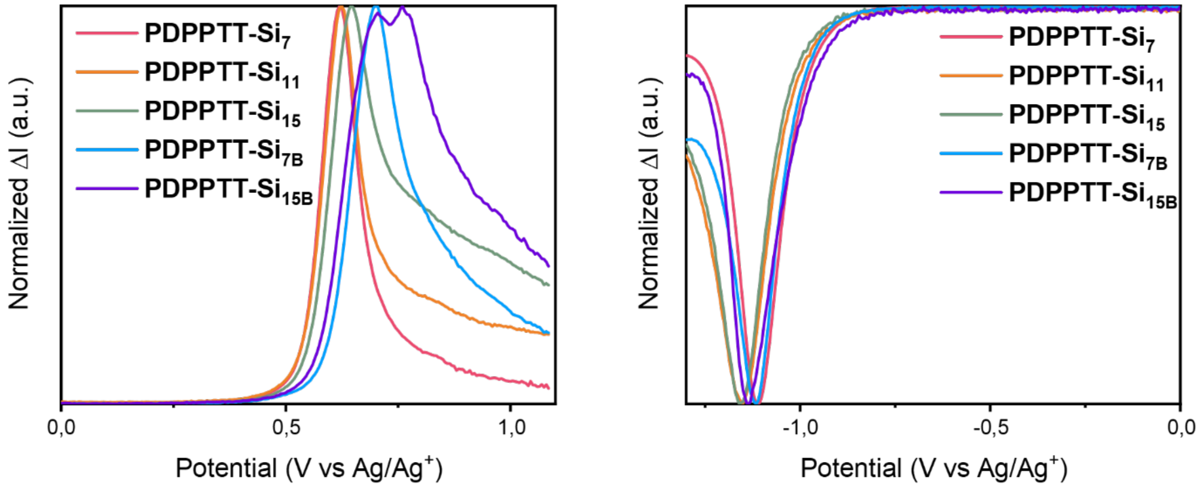

Figure S14: *Normalized square wave voltammograms of **PDPPTT-Si$_n$**.*

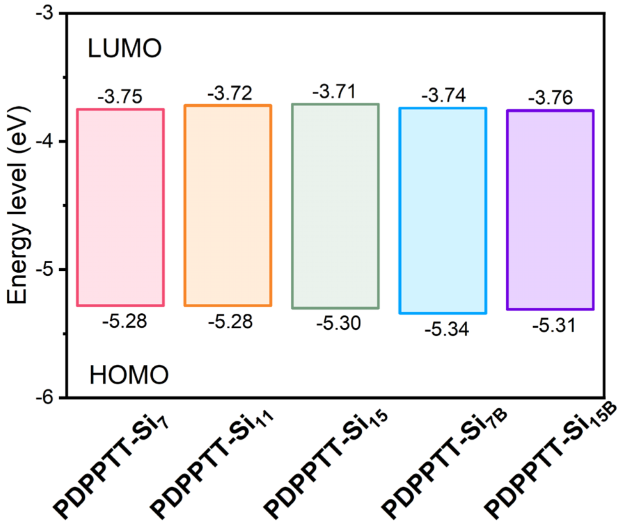

***Figure S15:** HOMO and LUMO energy levels of **PDPPTT-Si$_n$** determined with SWV using thin films on dip-coated Pt electrode in ACN with 0.1M tetrabutylammonium hexafluorophosphate as electrolyte. Ferrocene/ferrocenium (Fc/Fc+) was used as an internal standard.*

9. BGTC OFETs characteristics of PDPPTT-Si_n

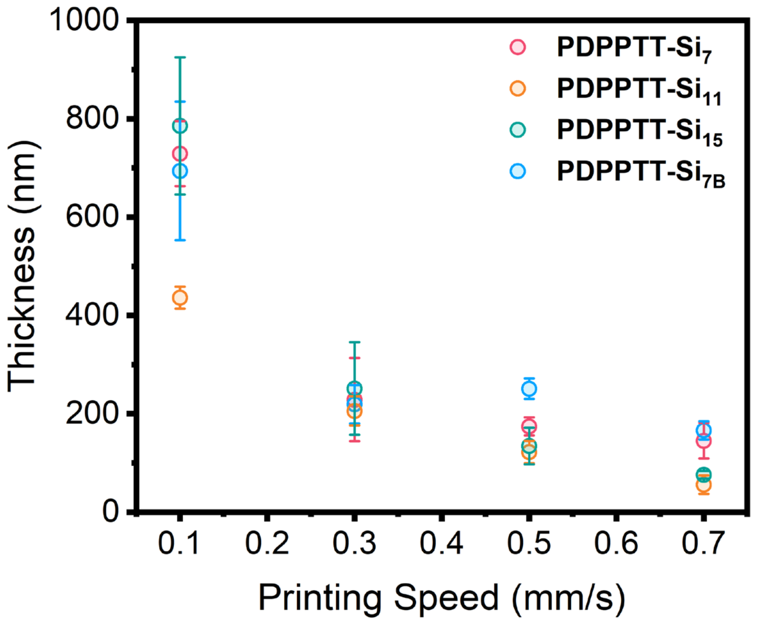

Figure S16: *Thickness data as a function of printing speed for the fabricated films.*

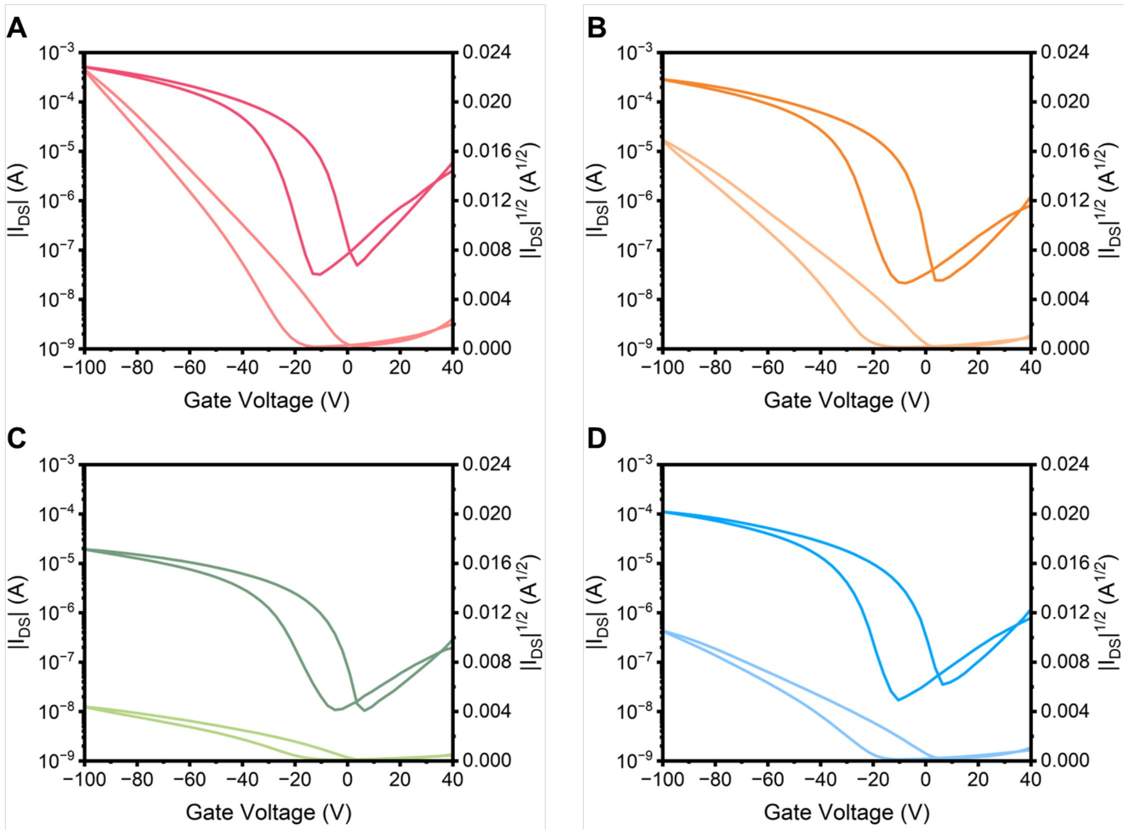

Figure S17: *Transfer curves for **PDPPTT-Si₇** (A), **PDPPTT-Si₁₁** (B), **PDPPTT-Si₁₅** (C) and **PDPPTT-Si₇B** (D). Transfer curves are shown as function of the drain current (dark) and root of the drain-source current (light).*

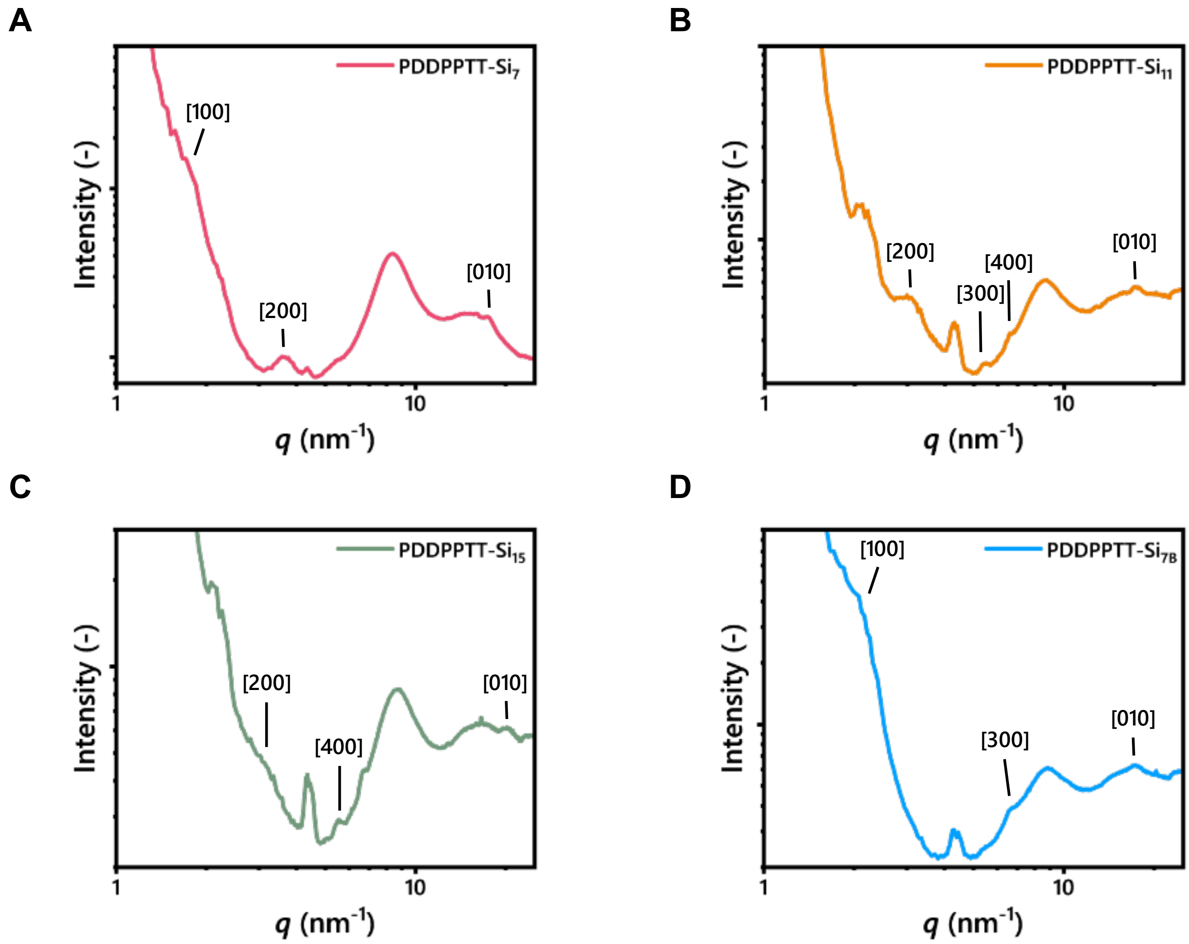

Figure S18: *1D GIXRD spectra of printed films of* ***PDPPTT-Si$_n$*** *recorded at φ = 0.080 showing lamellar ordering similar to previously observed in the bulk (Figure 3). Visible from the [010] reflection at 17.7 nm^{-1} for* ***PDPPTT-Si$_n$*** *is the π-π overlap with a distance of 3.55 Å.*

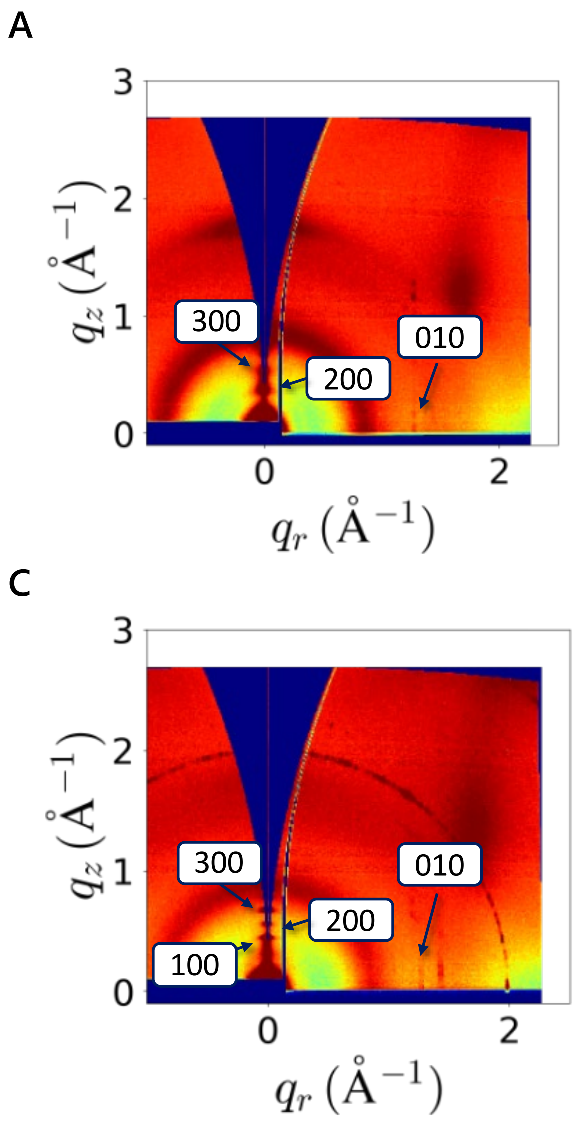

Figure S19: *2D GIXRD images recorded at φ = 0.080 of* ***PDPPTT-Si$_7$*** *(A),* ***PDPPTT-Si$_{11}$*** *(B),* ***PDPPTT-Si$_{15}$*** *(C) and* ***PDPPTT-Si$_{7B}$*** *(D). The concentration of the lamellar scattering reflections parallel to the q_r-axis is indicative of favorable edge-on orientation of the lamellae.*

10. NMR spectra

2,5-di(hex-5-en-1-yl)-3,6-di(thiophen-2-yl)-2,5-dihydropyrrolo[3,4-c]pyrrole-1,4-dione (1)

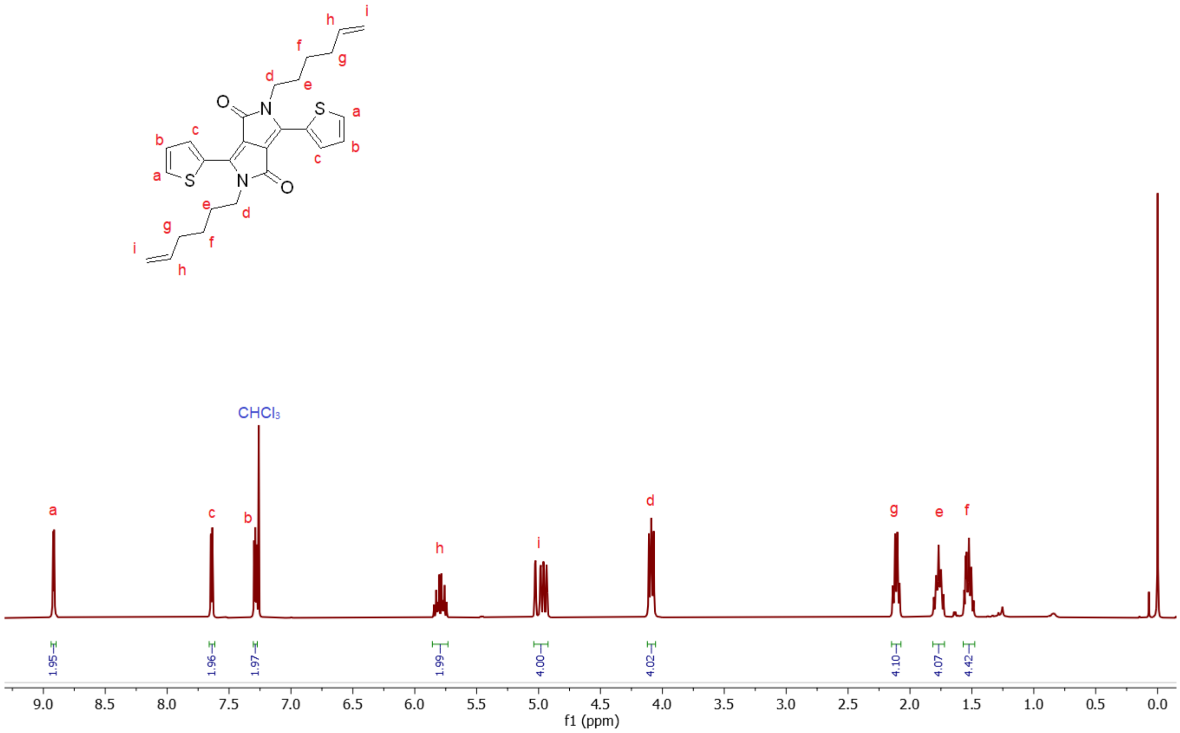

Figure S20: *^{1}H NMR (400 MHz, $CDCl_3$) spectrum of (**1**).*

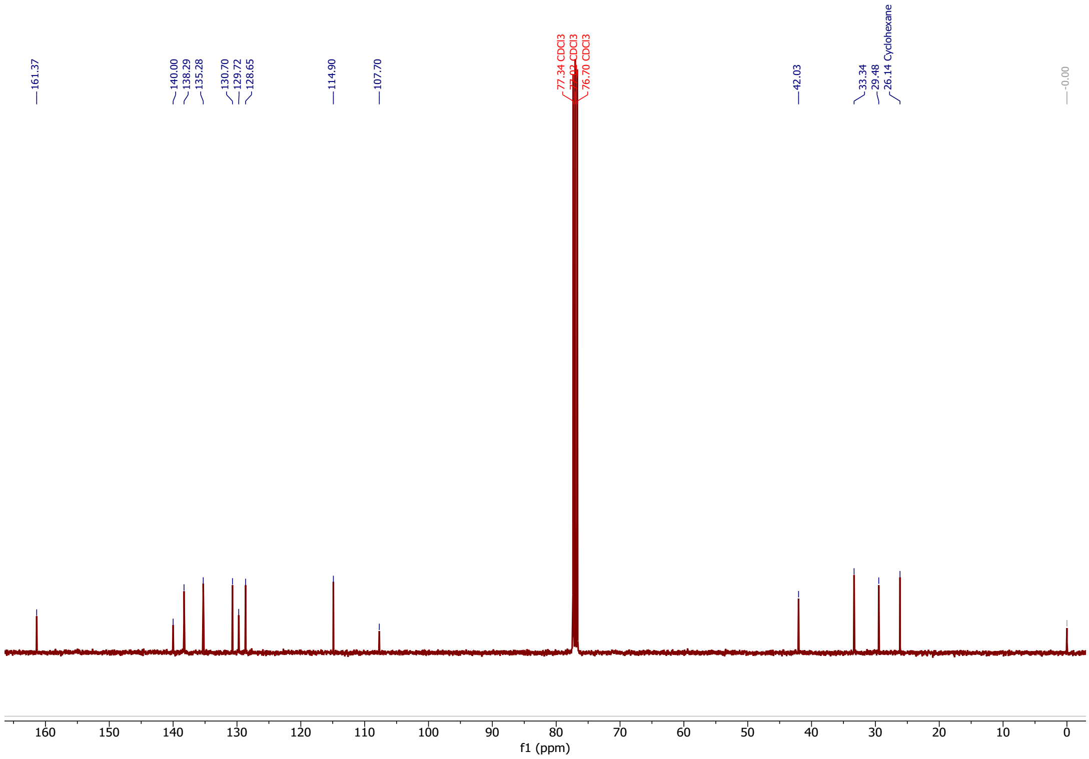

Figure S21: *^{13}C NMR (100 MHz, $CDCl_3$) spectrum of (**1**).*

2,5-bis(5-(1,1,3,3,5,5,7,7,9,9,11,11,13,13,13-pentadecamethylheptasiloxaneyl)pentyl)-3,6-di(thiophen-2-yl)-2,5-dihydropyrrolo[3,4-c]pyrrole-1,4-dione (2a)

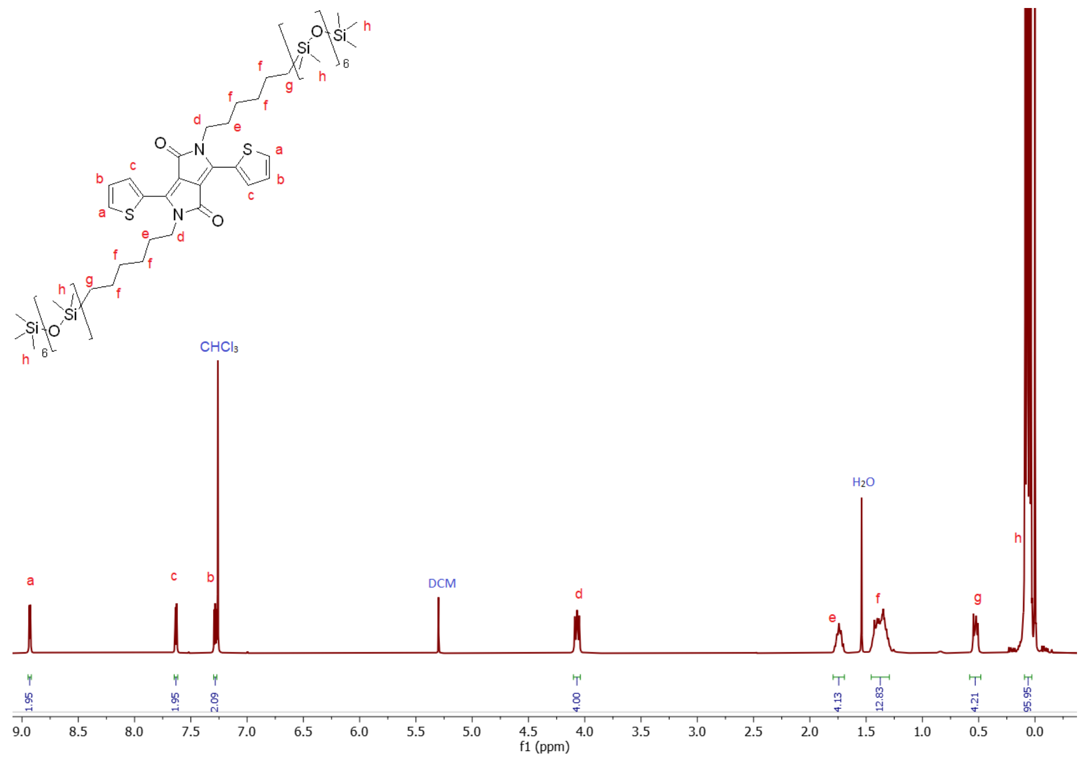

***Figure S22**: ^{1}H NMR (100 MHz, CDCl3) spectrum of (**2a**).*

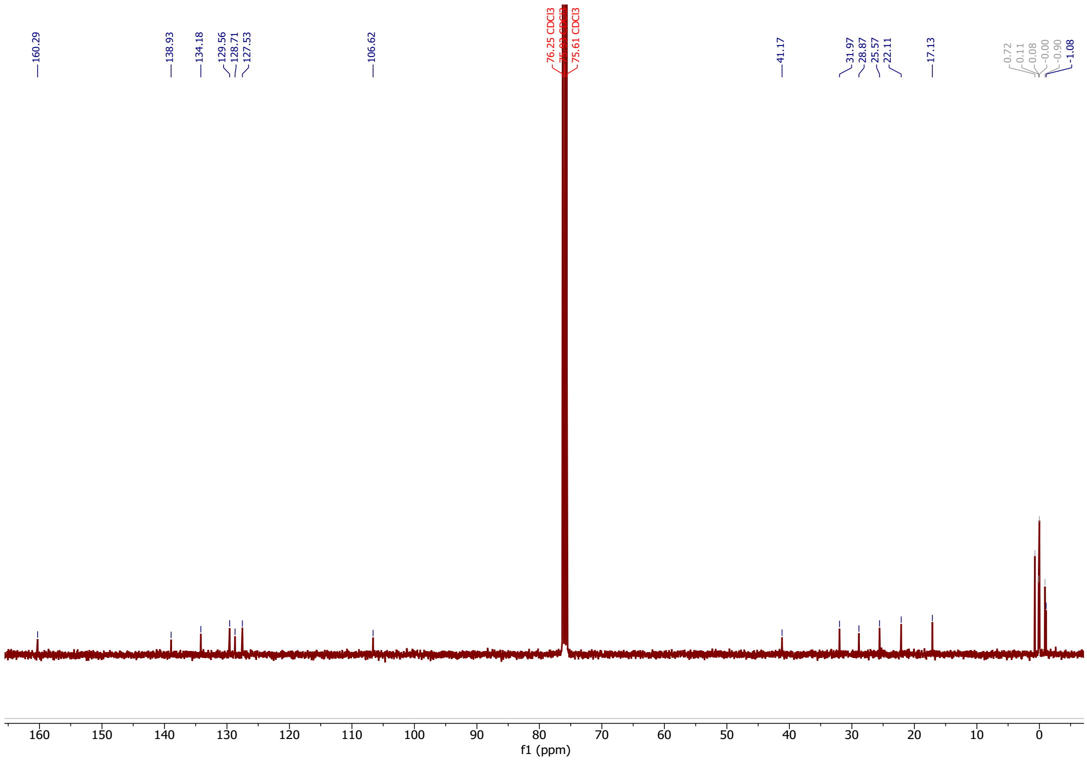

***Figure S23**: ^{13}C NMR (100 MHz, CDCl3) spectrum of (**2a**).*

3,6-di(thiophen-2-yl)-2,5-bis(6-(1,1,3,3,5,5,7,7,9,9,11,11,13,13,15,15,17,17,19,19,21,21,21-tricosamethylundecasiloxaneyl)hexyl)-2,5-dihydropyrrolo[3,4-c]pyrrole-1,4-dione (2b).

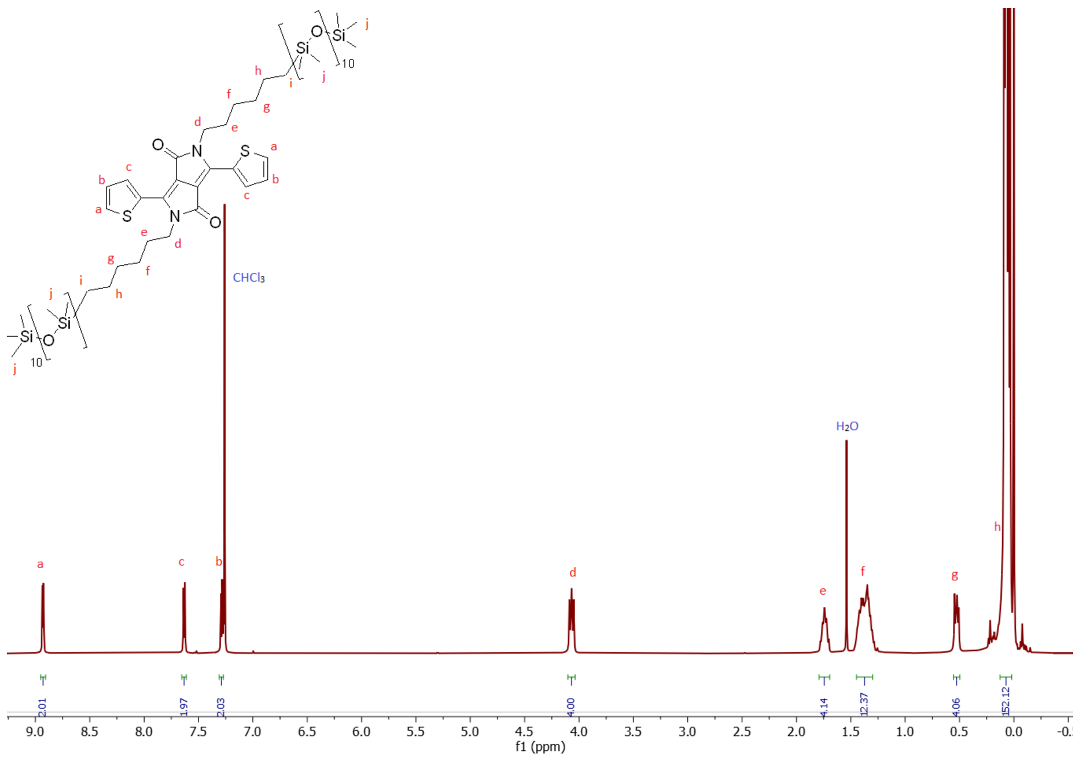

***Figure S24**: ¹H NMR (400 MHz, CDCl₃) spectrum of (**2b**).*

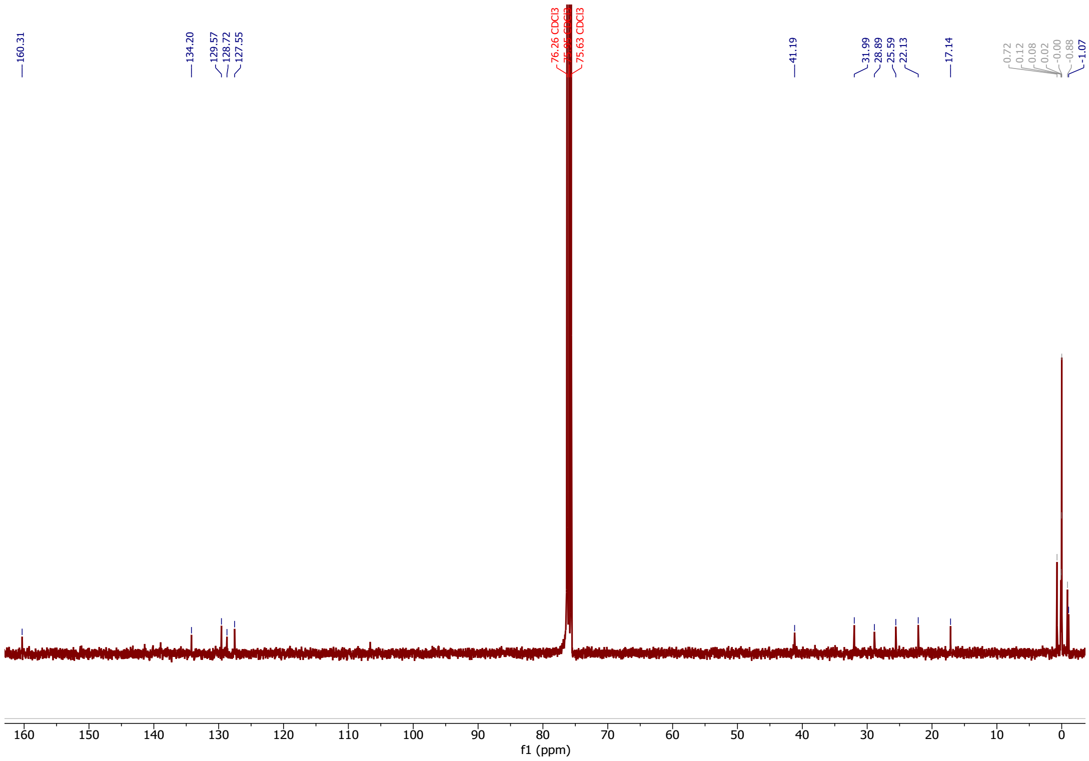

***Figure S25**: ¹³C NMR (100 MHz, CDCl₃) spectrum of (**2b**).*

2,5-bis(6-(1,1,3,3,5,5,7,7,9,9,11,11,13,13,15,15,17,17,19,19,21,21,23,23,25,25,27,27,29,29,29-hentriacontamethylpentadecasiloxaneyl)hexyl)-3,6-di(thiophen-2-yl)-2,5-dihydropyrrolo[3,4-c]pyrrole-1,4-dione (2c).

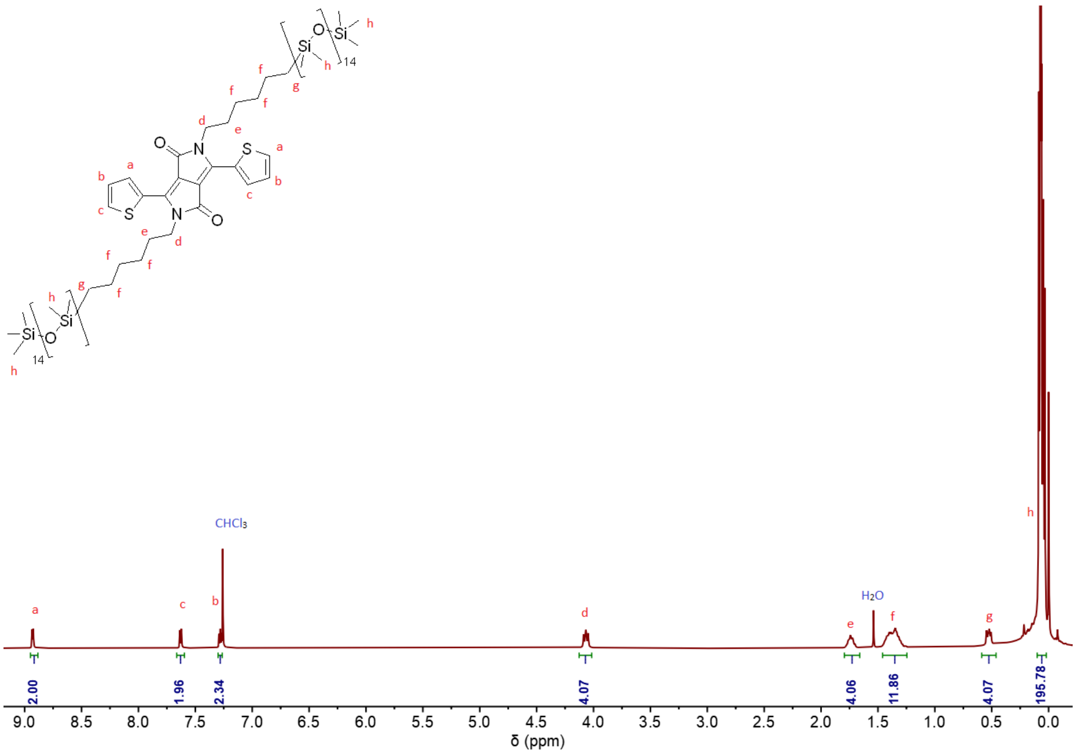

Figure S26: ^{1}H NMR (400 MHz, $CDCl_3$) spectrum of (**2c**).

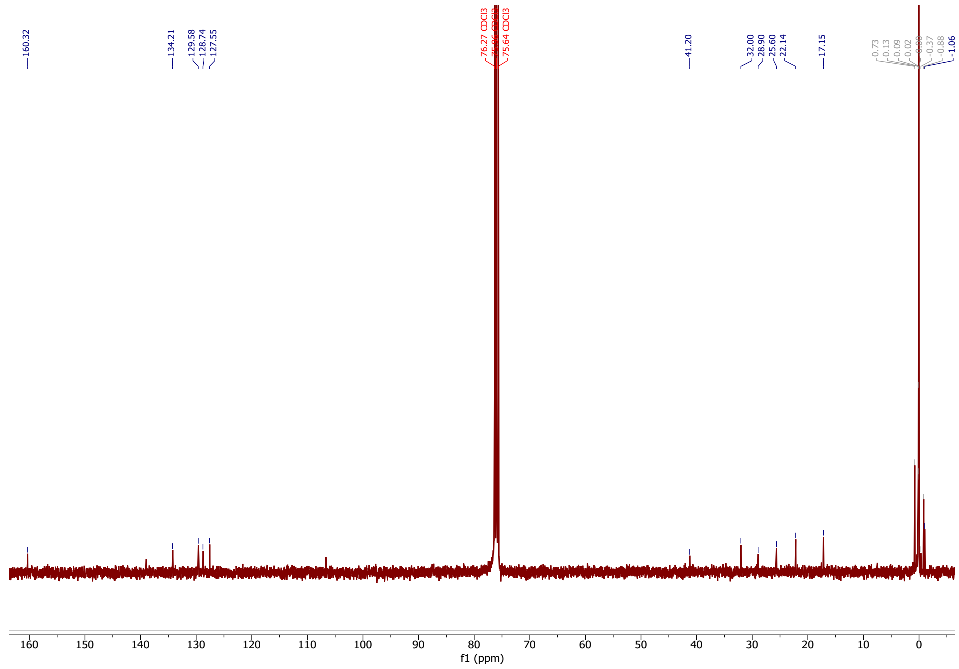

Figure S27: ^{13}C NMR (100 MHz, $CDCl_3$) spectrum of (**2c**).

2,5-bis(6-(1,1,1,3,3,5,5,7,9,9,11,11,13,13,13-pentadecamethylheptasiloxan-7-yl)hexyl)-3,6-di(thiophen-2-yl)-2,5-dihydropyrrolo[3,4-c]pyrrole-1,4-dione (2d).

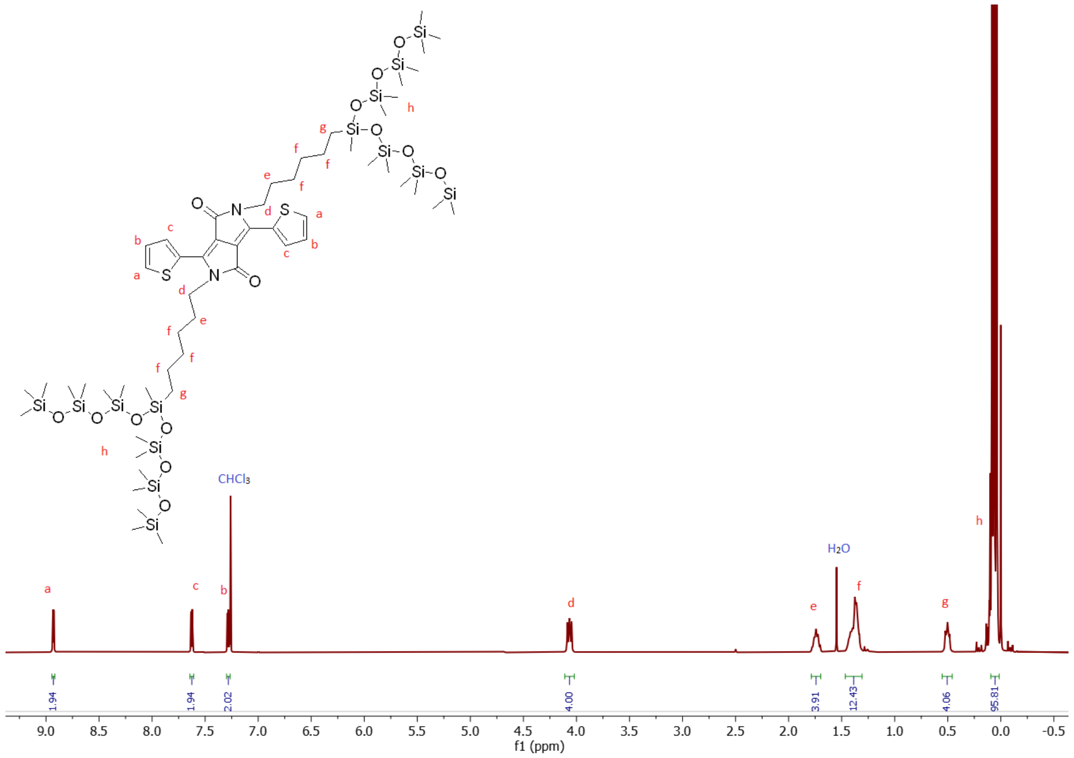

***Figure S28**: ^{1}H NMR (400 MHz, $CDCl_3$) spectrum of (**2d**).*

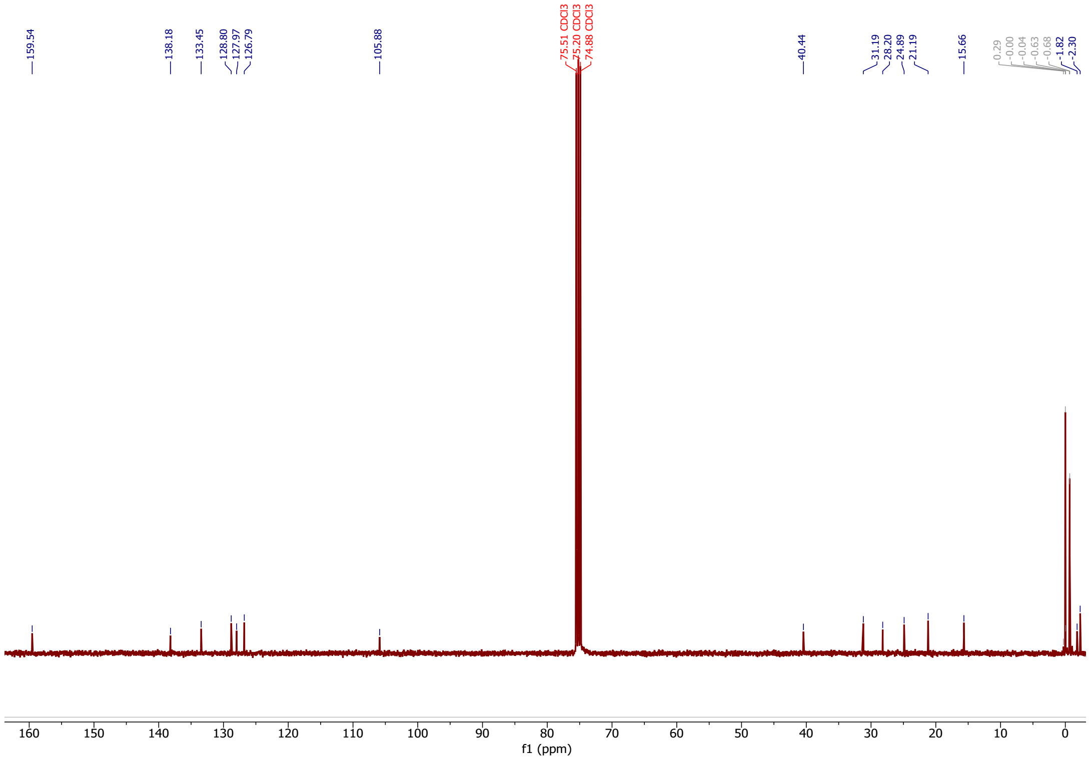

***Figure S29**: ^{13}C NMR (100 MHz, $CDCl_3$) spectrum of (**2d**).*

2,5-bis(6-(1,1,1,3,3,5,5,7,7,9,9,11,11,13,13,15,17,17,19,19,21,21,23,23,25,25,27,27, 29,29,29-hentriacontamethylpentadecasiloxan-15-yl)hexyl)-3,6-di(thiophen-2-yl)-2,5-dihydropyrrolo[3,4-c]pyrrole-1,4-dione (16).

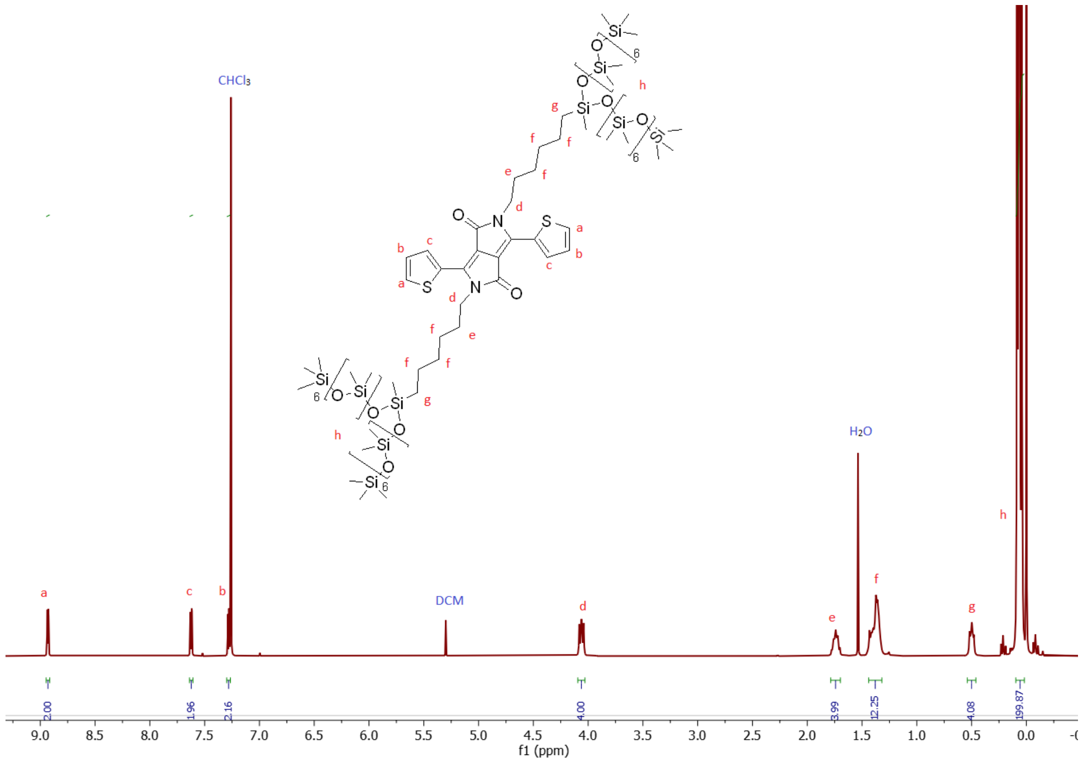

***Figure S30**: ^{1}H NMR (400 MHz, $CDCl_3$) spectrum of (**2e**).*

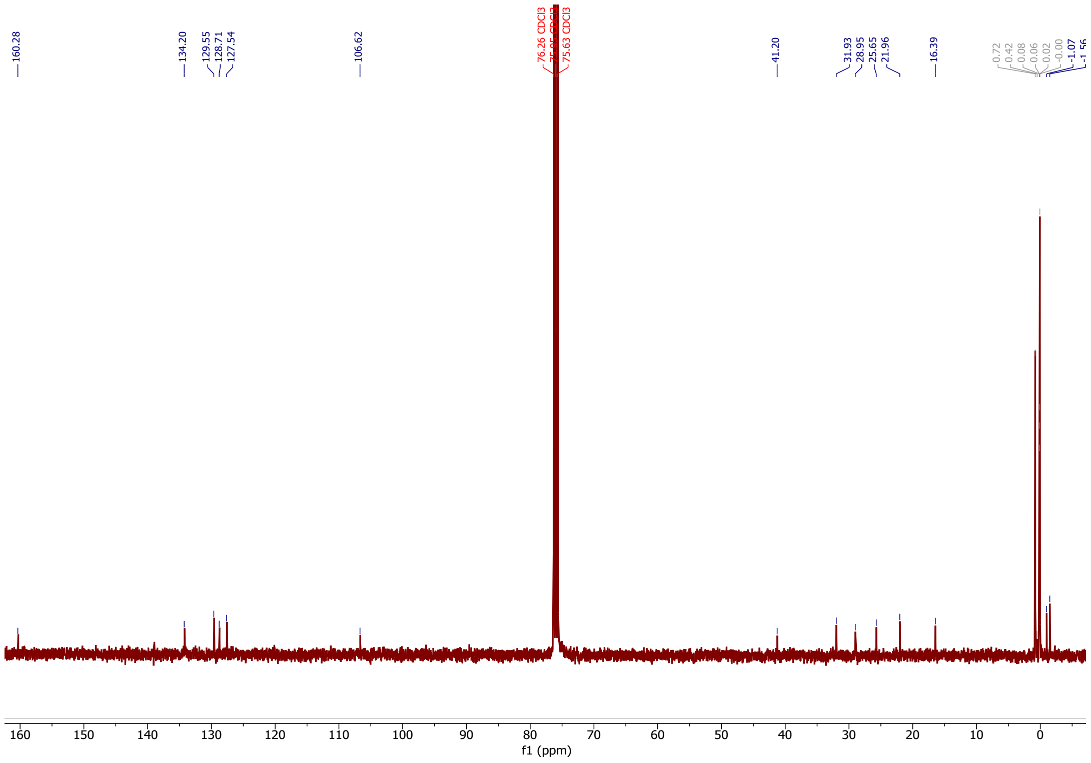

***Figure S31**: ^{13}C NMR (100 MHz, $CDCl_3$) spectrum of (**2e**).*

3,6-bis(5-bromothiophen-2-yl)-2,5-bis(6-(1,1,3,3,5,5,7,7,9,9,11,11,13,13,13-pentadecamethylheptasiloxaneyl)hexyl)-2,5-dihydropyrrolo[3,4-c]pyrrole-1,4-dione (3a).

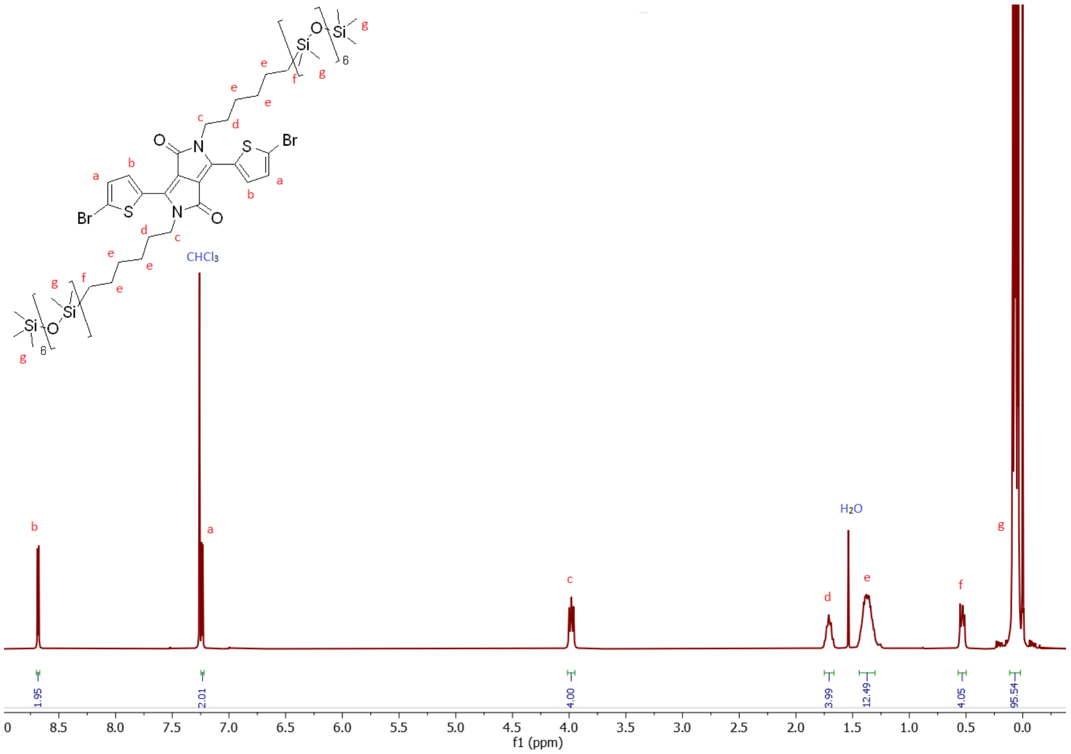

Figure S32: *^{1}H NMR (400 MHz, CDCl₃) spectrum of (**3a**).*

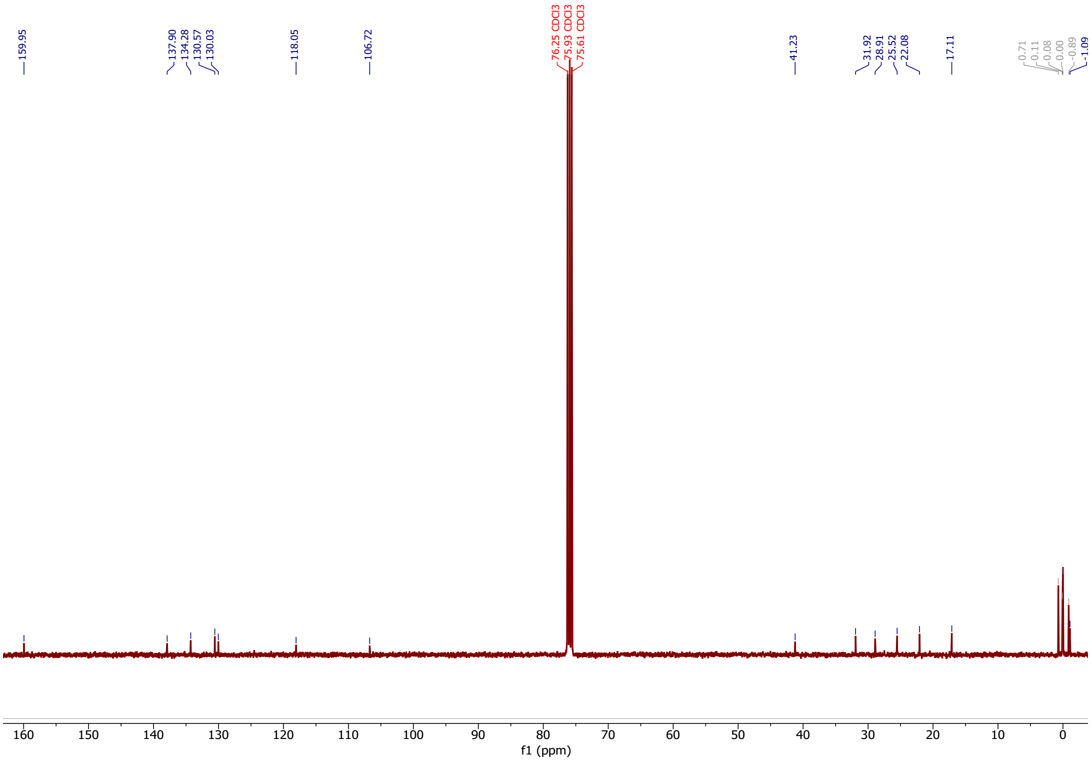

Figure S33: *^{13}C NMR (100 MHz, CDCl₃) spectrum of (**3a**).*

3,6-bis(5-bromothiophen-2-yl)-2,5-bis(6-(1,1,3,3,5,5,7,7,9,9,11,11,13,13,15,15,17,17,19,19,21,21,21-tricosamethylundecasiloxaneyl)hexyl)-2,5-dihydropyrrolo[3,4-c]pyrrole-1,4-dione (3b).

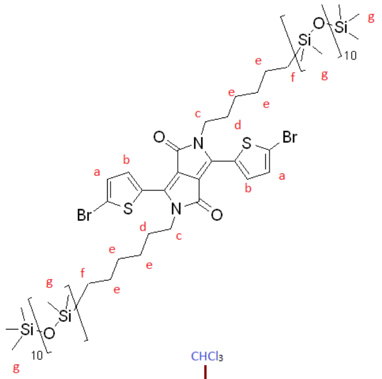

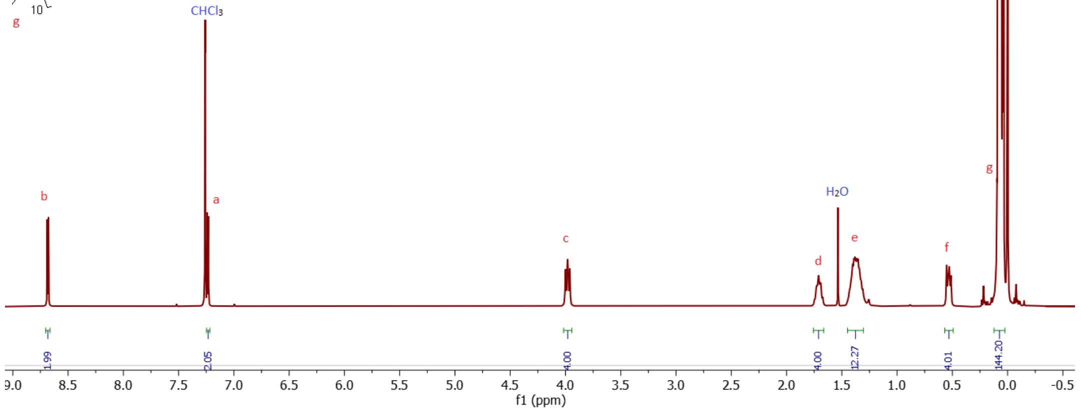

Figure S34: ^{1}H NMR (400 MHz, $CDCl_3$) spectrum of (**3b**).

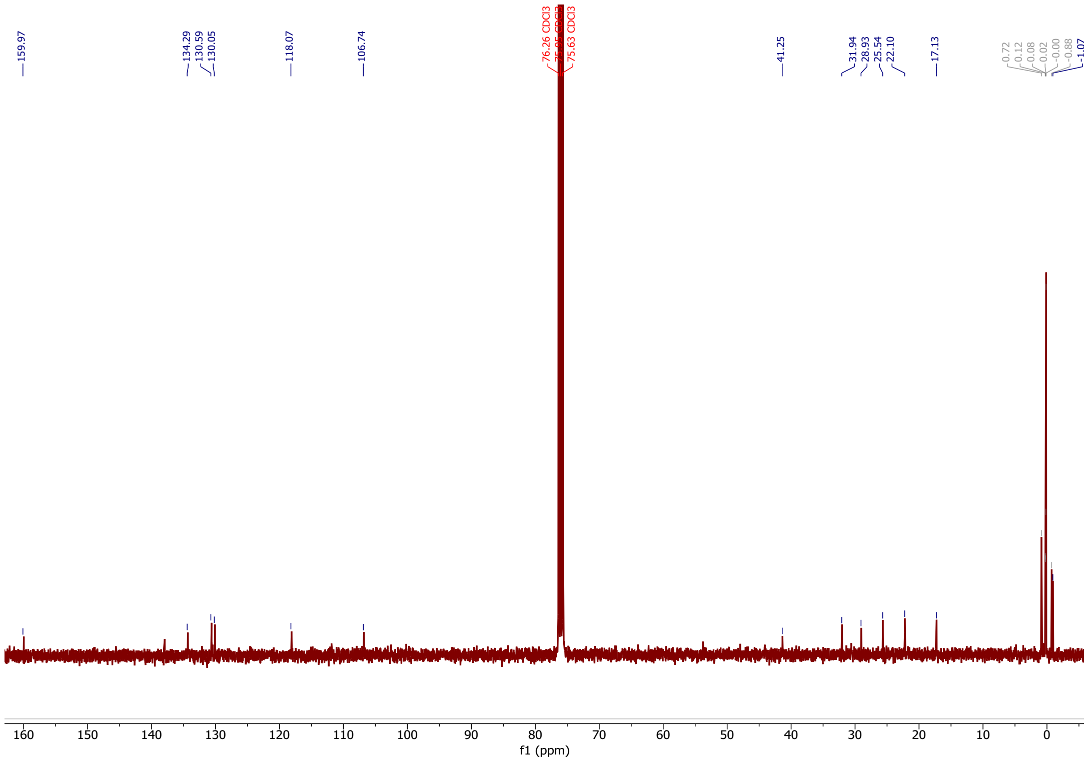

Figure S35: ^{13}C NMR (100 MHz, $CDCl_3$) spectrum of (**3b**).

3,6-bis(5-bromothiophen-2-yl)-2,5-bis(6-(1,1,3,3,5,5,7,7,9,9,11,11,13,13,15,15,17,17,19,19,21,21,23,23,25,25,27,27,29,29,29-hentriacontamethylpentadecasiloxaneyl)hexyl)-2,5-dihydropyrrolo[3,4-c]pyrrole-1,4-dione (3c).

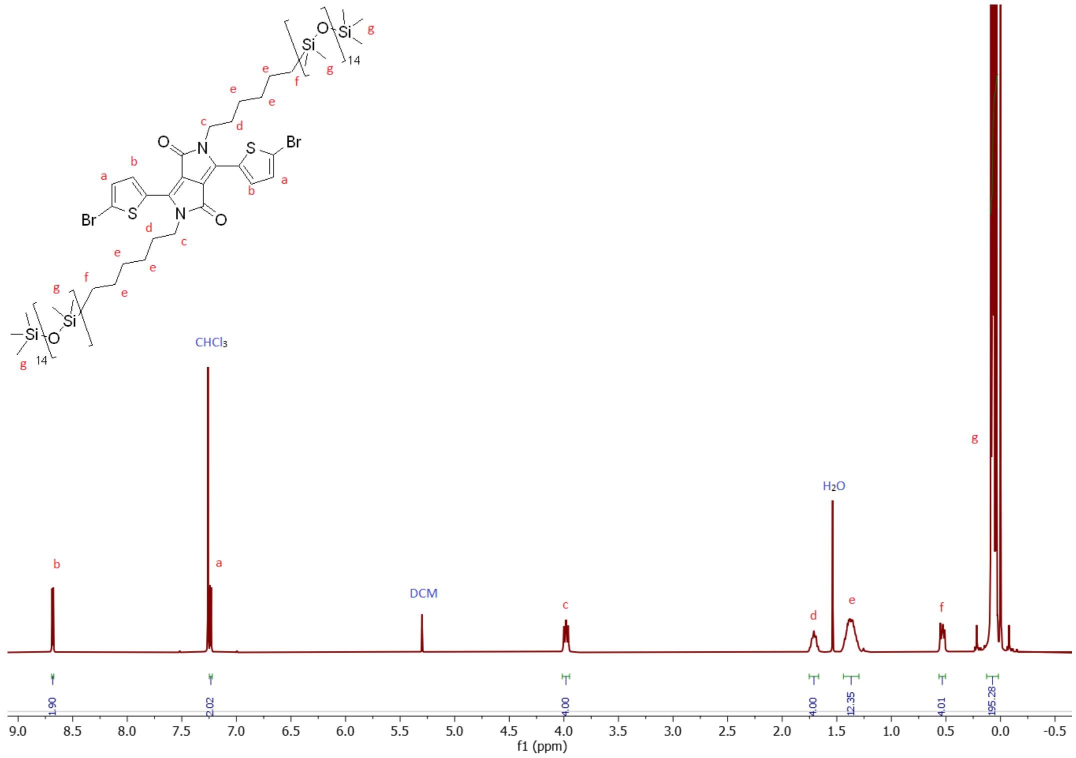

***Figure S36**: ¹H NMR (400 MHz, CDCl₃) of (**3c**).*

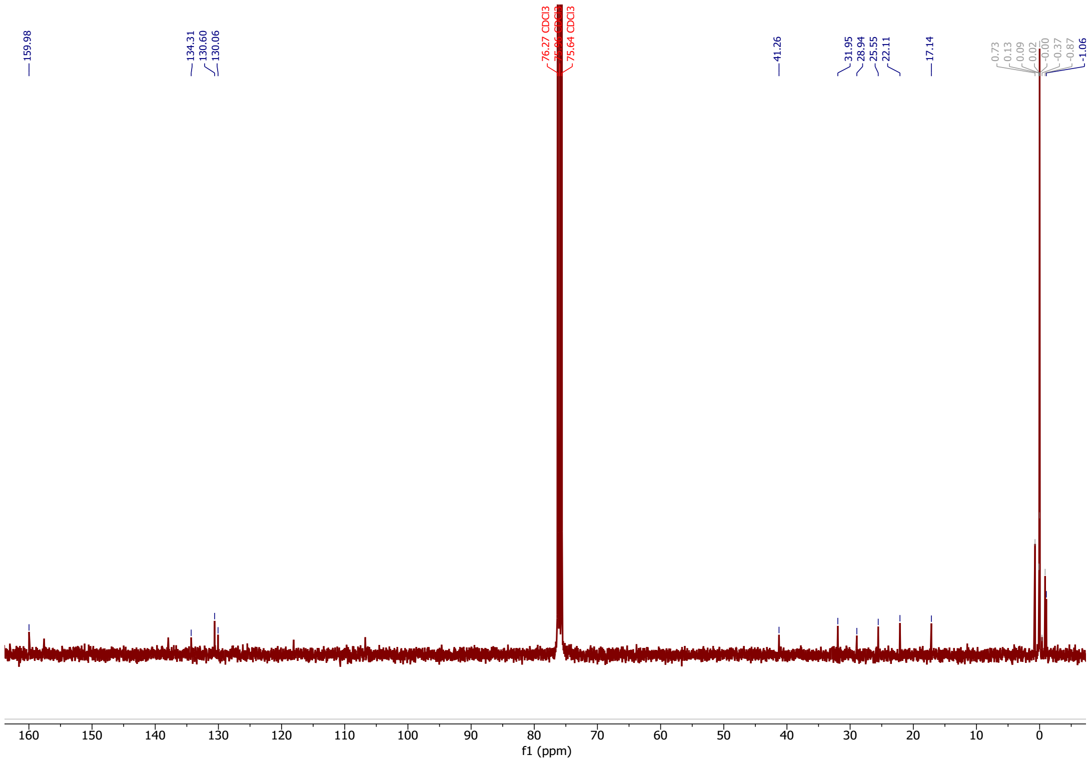

***Figure S37**: ¹³C NMR (100 MHz, CDCl₃) spectrum of (**3c**).*

3,6-bis(5-bromothiophen-2-yl)-2,5-bis(6-(1,1,1,3,3,5,5,7,9,9,11,11,13,13,13-pentadecamethylheptasiloxan-7-yl)hexyl)-2,5-dihydropyrrolo[3,4-c]pyrrole-1,4-dione (3d).

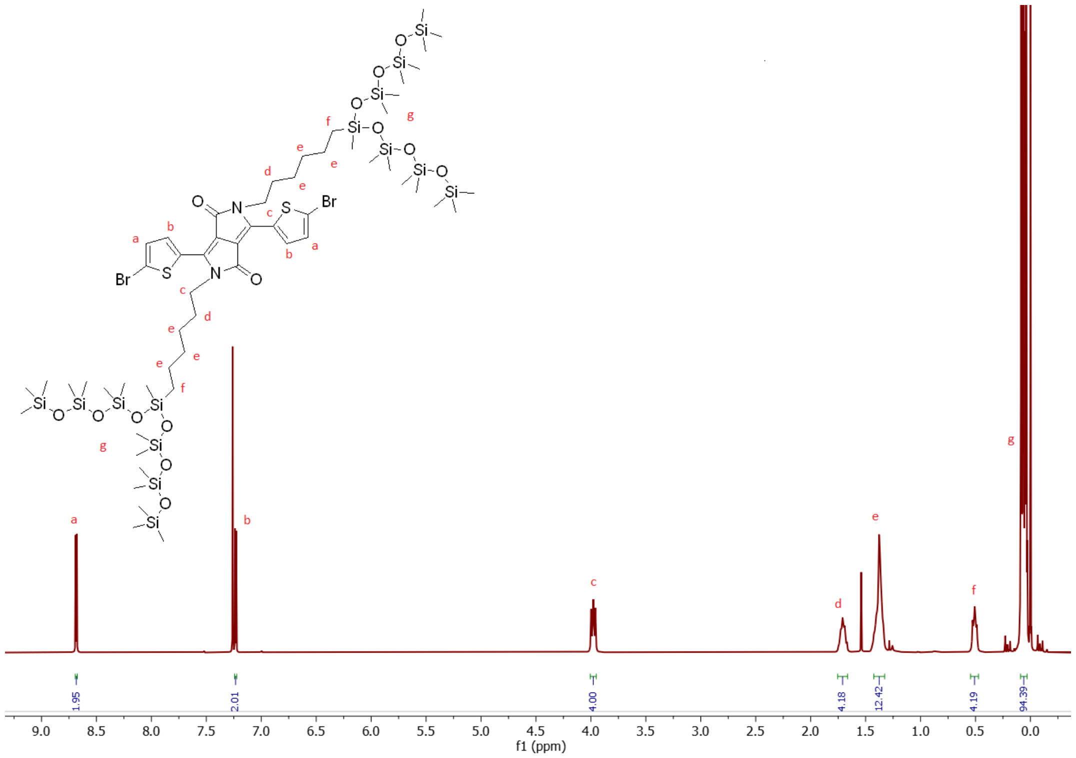

Figure S38: *^{1}H NMR (400 MHz, $CDCl_3$) spectrum of (**3d**).*

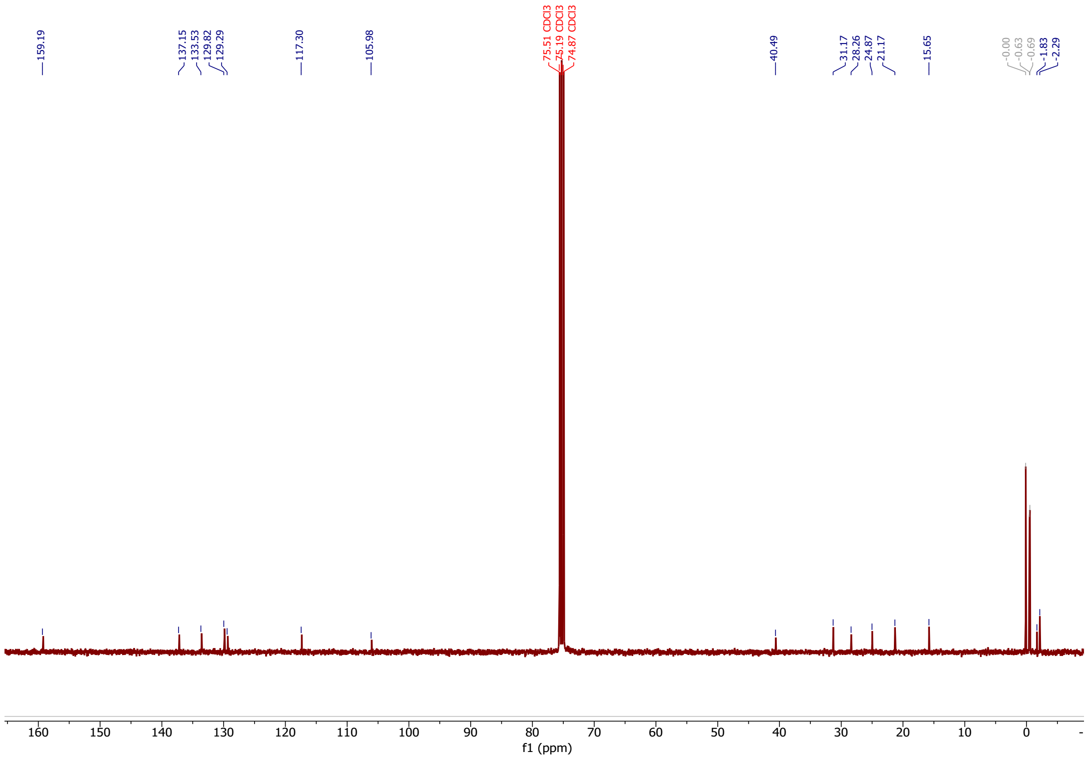

Figure S39: *^{13}C NMR (100 MHz, $CDCl_3$) spectrum of (**3d**).*

3,6-bis(5-bromothiophen-2-yl)-2,5-bis(6-(1,1,1,3,3,5,5,7,7,9,9,11,11,13,13,15,17,17,19,19,21,21,23,23,25,25,27,27,29,29,29-hentriacontamethylpentadecasiloxan-15-yl)hexyl)-2,5-dihydropyrrolo[3,4-c]pyrrole-1,4-dione (3e).

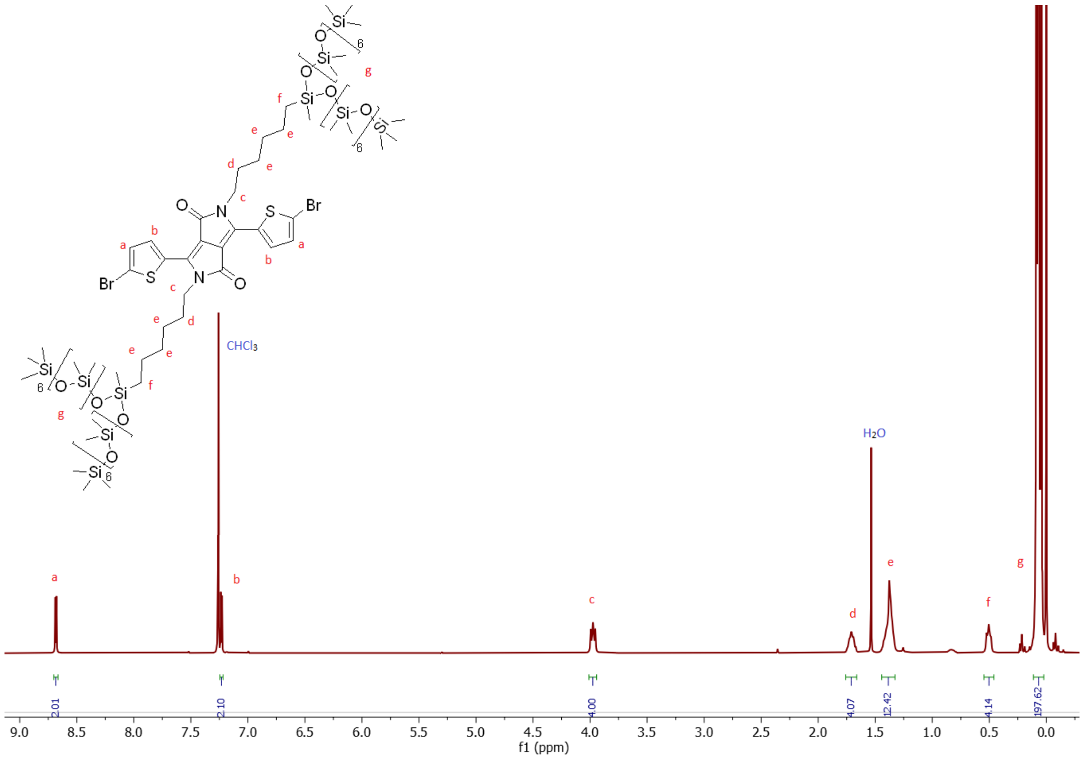

Figure S40: *^{1}H NMR (400 MHz, CDCl₃) spectrum of (**3e**).*

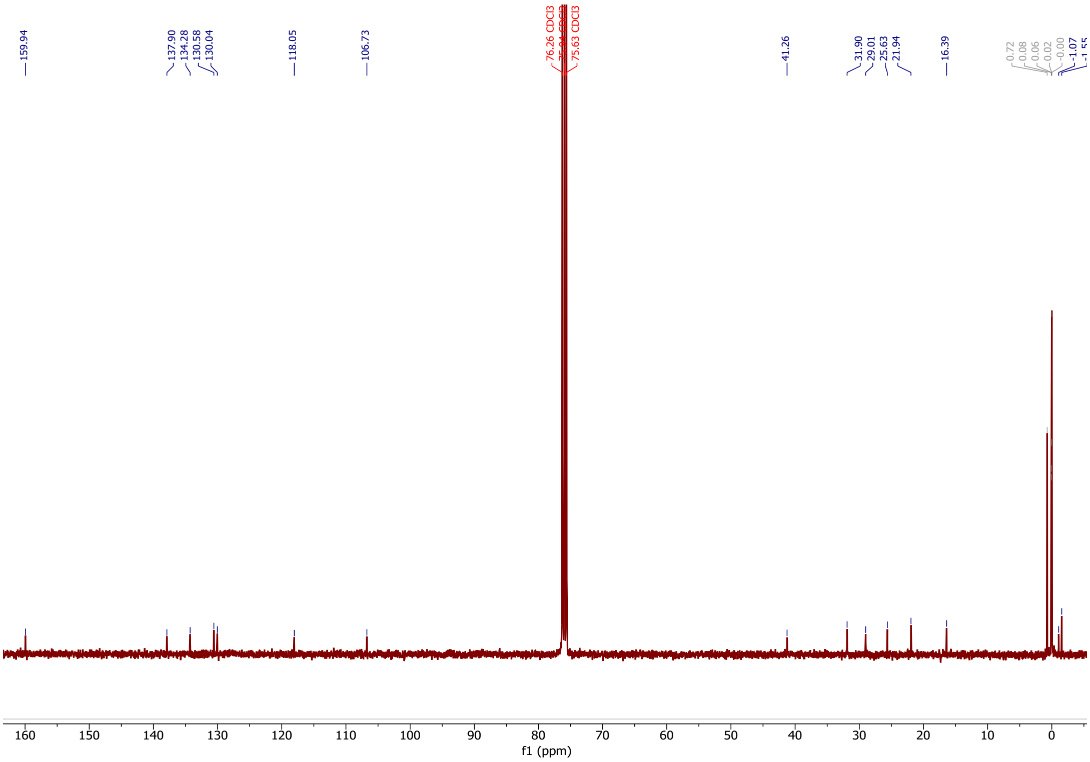

Figure S41: *^{13}C NMR (100 MHz, CDCl₃) spectrum of (**3e**).*

11. References

(1) Lamers, B. A. G.; Waal, B. F. M. de; Meijer, E. W. *J. Polym. Sci.* **2021**, *59* (12), 1142–1150.

(2) Van Son, M. H. C.; Berghuis, A. M.; De Waal, B. F. M.; Wenzel, F. A.; Kreger, K.; Schmidt, H.-W.; Rivas, J. G.; Vantomme, G.; Meijer, E. W. *Adv. Mater.* **2023**, *35* (25), 2300891.
